# Supplementary material for: Structural insights into i-motif DNA structures in sequences from the insulin-linked polymorphic region
Source: Nat Commun. 2024 Aug 20;15:7119. doi: 10.1038/s41467-024-50553-0 (PMC11336075; doi:10.1038/s41467-024-50553-0)
Supplement: Supplementary file 1 — Supplementary Information [file 41467_2024_50553_MOESM1_ESM.pdf]

SUPPLEMENTARY INFORMATION

Structural Insights into i-Motif DNA Structures in Sequences From the Insulin-Linked Polymorphic Region

Dilek Guneri,[1Ψ] Effrosyni Alexandrou,[1Ψ] El Omari Kamel,[2] Zuzana Dvořáková,[3] Rupesh V. Chikhale,[1] Daniel Pike,[1] Christopher A. Waudby,[1] Christopher J. Morris,[1*] Shozeb Haider,[1,4*] Gary N. Parkinson,[1*] Zoë A. E. Waller[1*]

1. School of Pharmacy, University College London, 29-39 Brunswick Square, London, WC1N 1AX, UK.
2. Diamond Light Source, Harwell Science and Innovation Campus, Chilton, Didcot OX11 0DE, UK.
3. Institute of Biophysics of the Czech Academy of Sciences, Královopolská 135, 612 00 Brno, Czech Republic.
4. UCL Centre for Advanced Research Computing, University College London, Gower Street, London WC1E 6BT.

[Ψ] These authors contributed equally to this work.

*z.waller@ucl.ac.uk, gary.parkinson@ucl.ac.uk, chris.morris@ucl.ac.uk, shozeb.haider@ucl.ac.uk

CONTENTS

1) SUPPLEMENTARY NOTE 1……………………………………………………………………………..PAGE 2
2) SUPPLEMENTARY NOTE 2……………………………………………………………………………..PAGE 10
3) SUPPLEMENTARY FIGURES…………………………………………………………………………..PAGE 16
4) SUPPLEMENTARY TABLES……………………………………………………………………………..PAGE 40
5) SUPPLEMENTARY REFERENCES……………………………………………………………………..PAGE 49

1) SUPPLEMENTARY NOTE 1

Supplementary results and discussion for enhanced sampling molecular dynamics simulations

The initial ILPR i-motif crystal structure consists of two near-identical motifs packed into a dimer as a result of crystallisation. Given the interactions between the sequences observed in the crystal structure originate from the flanking sequence, we looked at sequence 4C (TATCCCCACACCCCTATCCCCACACCCCTAT) and also an analogue with one base missing at the 3'-end of the flanking sequence, 4Cdel (TATCCCCACACCCCTATCCCCACACCCCTA).

The structures were prepared using the *tleap* module of the AmberTools20 package.[1] With regards to force-field parameters, a hybrid model, combining both bsc1 and OL15 modifications were used.[2, 3] As with all AMBER force-fields, these are additive and can be combined. Bsc1 is currently the best descriptor of alpha/gamma torsion angles, while OL15 best describes ε/**ζ** dihedrals and non-bonded interactions of bases. Further to these force-field parameters for protonated cytosine were taken from the AMBER force-field library: 'all_prot_nucleic10.lib'. In the case of the protonated structures, two of the four strands were protonated at the N3 position ($C_{4\text{-}7}$ and $C_{11\text{-}14}$), while the parameters mentioned previously apply to the remaining nucleotides.

Sufficient K^+ was added to the systems to neutralise the negatively charged phosphate backbones, as well as the C^+ atoms in the case of the protonated systems. Then excess K^+ and Cl^- atoms were added to simulate a 0.15 mol L^{-1} salt concentration. The systems were solvated using the TIP3P water model in an orthorhombic box, with all solute atoms at a minimum distance of 12 Å from the edge of the solvation box.

Each system was minimised and equilibrated under NVT conditions for 5 ns at 1 atm. The temperature was set to 300K using a timestep of 4 fs, all rigid bonds, a cut-off of 9 Å and particle mesh Ewald summation (PME) switched on for long-range electrostatics. The nucleic acid was initially fixed during equilibration, but these restraints were gradually removed, while the ions and solvent molecules were allowed to move. The heavy atoms of the nucleic acid were constrained by a spring constant set at 1 kcal mol^{-1} $Å^{-2}$. The production simulations were run in the NVT ensemble using a Langevin thermostat with a damping of 1.0 ps−1 and

hydrogen mass repartitioning scheme to achieve time steps of 4 fs. The AdaptiveBandit algorithm is implemented here,[4] and after each short run (50 ns) the algorithm is given a decision to select a pre-sampled conformation from which to restart the next short simulation - thus the objective is to avoid redundant sampling (i.e. visiting states which have already been sampled). The decision that is made aims to maximise a reward function. Here the reward function was defined to be proportional to relative phosphate-phosphate distances throughout the structure. The decision made each time aims to maximise the conformational space explored, using phosphate-phosphate distances as a metric.

The i-motif loops represent distinct sampling problems in that the core structure, stabilised by the hemi-protonated cytosine pairs, is largely stable with minor fluctuations. However, the loop regions are highly dynamic, able to sample multiple conformations with varying degrees of stability. The phosphate-phosphate distances from the structure were used as the Adaptive Bandit metric for the first 20 µs of simulation, after this only phosphate-phosphate distances in loop regions (8-10, 15-17, 22-24) were used as metrics. The loop-only sampling systems were run for a total of 30 µs. Thus, each system was run for a total sampling time of at least 50 µs.

As previously mentioned, i-motif loops represent a distinct sampling problem and conventional molecular dynamics simulations are not able to fully explore the conformational space in which it exists. This is overcome by use of adaptive sampling algorithms such as Adaptive Bandit, however the output from these many short simulations cannot be analysed directly therefore we need to integrate these simulations into Markov state models (MSMs). MSMs allow the integration of multiple simulation trajectories into a single model of the conformational landscape that contains both kinetic and thermodynamic information as well as maintain structural detail. MSMs are built on inter-state transitions; all of these states can be compiled and used to create a single model.

Here we used the PyEMMA package to create our models.[5] The general workflow for creating a Markov goes as follows. First, we use Time-lagged Independent Component Analysis (tICA) to reduce the dimensionality of the input feature. This differs from Principal Component Analysis in that it expands this reduced representation into the time domain. The data from tICA is then projected onto the first two (i.e. two slowest) ICs at a selected lag time and then clustered using the k-means algorithm. The number of clusters chosen represents the number

of clusters at which the VAMP2 scoring function no longer increases. This allows the numerous conformations to be mapped onto a set of discrete states. This then allows MSMs to be created that describe transitions that occur between any two clusters. The MSM calculates these transitions between states at a given lag time. It should be noted that this lag time is independent from that used in tICA. Choice of MSM lag time is key in that it must be sufficiently long to ensure Markovian dynamics (i.e. the system is memory-less and each transition isn't dependent on the previous state) but short enough to still be able to resolve the dynamics of the system. In order to choose this lag time we choose the smallest possible lag time at which the underlying processes (represented in an implied-timescale plot) show convergence. This model can then be validated using the Chapman-Kolmogorov test, which tests the ability of the model at longer time scales. The original space can then be discretized into the longest-living metastable states according to their eigenvectors. Then, the most stable structure is then extracted from each metastable state, as well as a distribution of 50 structures with an associated trajectory file to display transition between all 50 structures in a particular state. Finally, the PCCA++ algorithm is used to coarse-grain the conformational space of the metastable states and then approximate the stationary distribution of the data as well as approximating the relative free energies. A total of 6 Markov state models were created to describe different aspects of the dynamics of the i-motif structure, details of which are displayed in Table S10. A combination of α/γ angles and the distance between N3 atoms of the stem cytosines were used as features to describe the central stem, while a combination of α/γ angles and χ angles were used as features to describe the dynamics of the loops.

In the ILPR i-motif structure there are three loops, 1 ($A_8C_9A_{10}$) and 3 ($A_{22}C_{23}A_{24}$) are distal to the 5' and 3' ends, while loop 2 ($T_{15}A_{16}T_{17}$) lies in the middle at the sequence and is flanked in space by both the 5' and 3' prime ends. 4Cdel, loop 1 consists of C_9 protruding away from the centre of the structure and into the solvent, while A_{10} intercalates between A_8 and C_7, interacting with both via π-stacking, as well as forming a non-canonical base pair with A_{22}. In loop 3, A_{22} is pointed towards the centre of the structure and caps C_{21}, while C_{23} and C_{24} protrude into the solvent (as a result of crystal packing) with their faces around 6.5 Å apart. In loop 2, T_{17} interacts with T_3 via two hydrogen bonds formed between (O4 and N3), this pair forms in parallel to the C:C$^+$ pair above and stabilises these via π-π interactions. T_{17} itself is

stabilised via π-stacking with T_{16} below. T_{15} forms no obvious actions and seems to shield the internal structure of the i-motif from solvent. The 4C structure is near-identical apart from A_{10}, which instead of intercalating between A_8 and C_7, is flipped outward into the solvent, A_8 instead, forms the non-canonical base pair with A_{22}. This is also the case with A_{16}, which protrudes into the solvent instead of stacking below $T_{17,}$ which itself is instead stabilised from below by T_{29}.

In order to assess the individual components that contribute to structural stability within the i-motif, MSMs were created with features extracted from only loop regions (bases 8-10, 15-17 & 22-24) and then with features from stem regions of the structure (bases 4-7, 11-14, 18-21). As expected, the loop regions are far more flexible and the majority of dynamic motion within the structure can be attributed to these regions. This is borne out in the fact that the stem regions required significantly larger tICA (40 ns) and MSM lag times, than the models of the loop systems, to fully describe the conformational landscape. This is also displayed by the findings that the stem only exists in a two-state system, displayed by a double-well potential (Figure S18/19). These two states have an RMSD < 1.2 Å, whereas the conformational landscapes are split into many more metastable states with significantly higher RMSD values. Although not a perfect comparison, this agrees with similar work on G-quadruplex structures using similar force-fields, as such validating the fact that these are suitable to study i-motif stem structures and $C:C^+$ pairs.

Sampling of the crystal structure conformations

The crystalline unit cell consists of two individual strands related by an inverted symmetry. The two strands (4C and 4Cdel from here onwards) are linked with the 5' end of each unit interacting with loop 3 of the other. However, the interactions in these regions are not identical; A_2 of strand 4C is able to form two hydrogen bonds with A_{24} in strand B due to a slight misalignment this interaction does not occur in the analogous region in the inverted symmetry. However, T_1 stabilises A_{24} via a π-stacking interaction in both cases.

The ability of a simulation to fully sample its starting structure is often used as a benchmark to validate the accuracy of the sampled conformation. In our case of the ILPR i-motif, several favourable interactions occur as a result of crystal packing and therefore the conformation found within the crystal structure may not be a realistic depiction of the structure *in vivo*.

Given that once these interactions are removed, they are likely to be compensated by other interactions. Thus, the crystal structure can no longer be defined as the most stable state and may rarely be visited throughout the course of a simulation. This is particularly relevant in the case of loop 3 where crystal packing effects lead to complete solvent exposure when the structures formed in the crystalline lattice are separated. Still, assessing whether the conformations adopted by the loops, during the simulations, are able to sample their crystal structure conformation is nevertheless a useful benchmark to assess the accuracy and quality of the model created. All the loops are able to sample conformations observed in the crystalline lattice in spite of the loss of crystal packing interactions. For example, A_8-A_{10} (loop 1), A_{16}-T_{17} (loop 2) and A_{22}-C_{21} (loop 3) π-stacking interactions in strand A and C_7-A_8 (loop1), T_{15} π-stacking below T_3:T_{17} base pair and C_{21}-A_{22} (loop3) π-stacking interactions in strand B are all observed (Figure S20).

Loop Dynamics

Given the intrinsic rigidity of the i-motif stems enforced by the hydrogen bonding between cytosines and repulsive forces between neighbouring carbonyl and amino groups in the $C:C^+$ pair stacks, the regions either side of the stem (loop 1/3 and loop 2) can be viewed as a two different sub-systems as the dynamics in one region is likely to have a minimal effect on that of the other. Therefore, in order to assess loop conformations more directly, MSMs were created for the sequences 4C and 4Cdel in which only loop features were used as inputs for tICA, namely α/γ angles and χ angles which have proven useful previously to assess loop dynamics in G-quadruplexes.

Loop 2

In 4C, the most stable conformation (state 4) represents 86% of all sampled conformations. This conformation samples the interactions observed in the crystal structure including the T_3:T_{17} base pair. Stacked below the T_3:T_{17} base pair is the T_5:A_2:T_{29} triad. In the triad, T_{15} is slightly off plane. Stacking directly below A_2:T_{29} is T_1:A_{30} base pair. The 3' end terminal T_{33} stacks below A_{30}. A_{16} from loop 2 is solvent exposed and interacts with the 3' hydroxyl group

of T33 (Figure S21). These series of base stacks between the flanking termini and loop 2 help to stabilise the structure. In 4Cdel, which is missing the terminal T in the sequence, loop 2 dynamics can be seen as the interconversion between four primary states as illustrated in the free energy landscape (Figure S22).

The most stable conformation (state 5), representing 39% of the equilibrium distribution system, perfectly samples the conformation displayed in the crystal structures; namely T_{15} tilted inwards, T_3 in plane with T_{17} to form a non-canonical T:T base pair, with two hydrogen bonds formed between O4 and N3 atoms that stabilises the C:C+ pair above, as well A_{16} stacking below it. Interestingly T_2 from the 5′ end makes a canonical base pair with A_{16}, to add further stability to the T_3:T_{17} base pair. Finally, and as expected when crystal packing effects are removed T_{29} and A_{30} fold inwards and stack together (Figure S23).

When comparing the dynamics of loop 2 between sequences 4C and 4Cdel, it becomes evident that 4C is significantly more skewed towards a single structure with metastable state 4 representing 86% of the equilibrium population. This skewing might be a result of the longer 3′ end which leads to enhanced π-stacking and hydrogen bond interactions with loop 2, and thus leading to greater stability. The elongated 3′ end also presents a more plausible version of what may happen *in vivo* as there are likely to be at least three residues between the start/end of the i-motif and the start of other canonical/non-canonical nucleic acid structures. It is also worth noting that the position and orientation of T_{15} is unchanged across the metastable structures, highlighting to its importance in maintaining structural integrity within this region. Given its position and orientation and the interactions it makes with A_2, it is likely that A_{15} acts like a hydrophobic cap that impedes the entrance of water into the core of the structure and therefore likely interference with the C·C^+ pair. Finally, our results validate the importance of non-canonical base pairs acting as capping residues in i-motif structures. All structures contain a T:T base pair between T_3 and T_{17} that is analogous to that of the C:C^+ pair and thus extends the loop and further stabilised the terminal pair of the stem through favourable stacking of exocyclic carbonyls and amino groups in an antiparallel manner.

Loops 1 and 3

To assess the dynamics of loops of 1 and 3 only, without the models being affected by the dynamics around loop 2, MSMs were then created using only the α/γ angles and χ angles of loop 1 and 3 only as they act as a singular interacting system and their dynamics should be taken together into account. The conformational space of loops 1 and 3 in strand A can be described as the interconversion between 6 metastable states (Figure S24). The most populated state is state 6 accounting for 52% of all sampled conformations. In loop 1, A_{10} is able to stack on C_7 to form a non-canonical base pair interaction with A_{22}, with hydrogen bonds formed between the N6 and N7 atoms in both bases. Interestingly, in this cluster we observe stacking of C_9 above A_{22} and A_8 above A_{10}. Metastable state 6 recapitulates the conformations and interaction that are observed in the crystal structure (Figure S20). State 3 is a substate of state 6, where C_9 stacks above A_{10}:A_{22} base pair. In metastable state 5 (38% population), the A_{10}:A_{22} base pair is formed but there is no stacking observed by A_8 or C_9 bases above the A_{10}:A_{22} base pair. State 4 is a substate of metastable state 5, where the A_{10}:A_{22} base pair is not formed, while A_8 makes π-stacking with A_{10}. State 1 is a low populated, high energy conformation where loop 1 and 3 are collapsed on the terminal C_7:C_{21} base pair. The A_{10}:A_{22} base pair that stacks on top of C_7:C_{21} base pair is off centred, while C_9 stacks on top of A_{10}:A_{22} base pair instead of A8, which is tucked in a groove and is shielded from the solvent by A_{24}. Nucleotide A_{23} is solvent exposed. State 2 population represents the open conformation of the loop 1 and 3. In this state, the loops move outward exposing the A_{10}:A_{22} base pair and in some instances the C_7:C_{21} base pairs.

Loop 1 and 3 in 4C are separated by tICA into 4 metastable states (Figure S25). State 1 in 4C represents the crystalline conformation where A_8:A_{22} base pair is present, while bases C_9, A_{10}, C_{23} and A_{24} are solvent exposed. In state 2, the A_8 and A_{22} base pair is lost and the sugar phosphate backbone of loops 1 and 3 is in a constrained conformation pointing towards the central i-motif stem. Unlike 4Cdel, loop 1 in the most populated state 3 (91%) of 4C does not fully sample the conformation found in the crystal structure. The A_8:A_{22} base pair is abolished and A_{22} is instead stabilised by forming two hydrogen bonds with its N6 amine, making interactions with the O4' and N3 atoms in A_{10}. A_8 is stabilised through the contact between its N6 amine and the phosphate oxygen in A_{24}. A_{23} and A_{24} stack next to each other in a vertical

orientation, this stacking arrangement is further stabilised by the interaction between the A_{24} N6 amine and the C_{23} phosphate group. Furthermore, the slight inward rotation of loop 3 allows the stabilisation of the C_7 N4 amine via interaction with the A_{24} phosphate. State 4 is similar to state 3. The only difference is the arrangement of C_9, A_{10}, C_{23} and A_{24} bases in loops 1 and 3 respectively. To summarise, upon inspection of structures extracted from coarse-grained models, 4Cdel featured far more unstructured conformations in loops 1 and 3 while those in 4C, with the full flanking sequence, are well ordered.

2) SUPPLEMENTARY NOTE 2

Sanger sequencing of sub-cloned INS421 plasmid with different ILPR inserts

The FASTA format of the sequences is shown after processing of Sanger sequencing as following; lowercase letters are the original pGL410_INS421 firefly expression plasmid with uppercase letters representing the human insulin promotor (a gift from Kevin Ferreri via Addgene; plasmid #49057; http://n2t.net/addgene:49057; RRID:Addgene_49057)[6] underlined and bold letters represent identifying restriction digest site for ILPR variant confirmation; bold and uppercase letters are showing the selected ILPR variants for reporter gene assay.

ILPR 1G/C

Ggcctaactggcc**ggtac**ctGAGC**TGTGGGGACAGGGGTGTGGGGACAGGGGTGTGGGGACA**GCAGCG
CAAAGAGCCCCGCCCTGCAGCCTCCAGCTCTCCTGGTCTAATGTGGAAAGTGGCCCAGGTGAGGGC
TTTGCTCTCCTGGAGACATTTGCCCCCAGCTGTGAGCAGGGACAGGTCTGGCCACCGGGCCCCTGGT
TAAGACTCTAATGACCCGCTGGTCCTGAGGAAGAGGTGCTGACGACCAAGGAGATCTTCCCACAGA
CCCAGCACCAGGGAAATGGTCCGGAAATTGCAGCCTCAGCCCCCAGCCATCTGCCGACCCCCCCACC
CCAGGCCCTAATGGGCCAGGCGGCAGGGGTTGAcAGGTAGGGGAGATGGGCTCTGAGACTATAAA
GCCAGCGGGGGCCCAGCAGCCCTCAGCCCTCCAGGACAGGCTGCATCAGAAGCTTggcaatccggtact
gttggtaaagccaccatggaagatgccaaaaacattaagaagggcccagcgccattctacccactcgaagacgggaccgccggcg
agcagctgcacaaagccatgaagcgctacgccctggtgcccggcaccatcgcctttaccgacgcacatatcgaggtggacattacc
tacgccgagtacttcgagatgagcgttcggctggcagaagctatgaagcgctatgggctgaatacaaaccatcggatcgtggtgtg
cagcgagaatagcttgcagttcttcatgcccgtgttgggtgccctgttcatcggtgtggctgtggccccagctaacgacatctacaac
gagcgcgagctgctgaacagcatgggcatcagccagcccaccgtcgtattcgtgagcaagaaagggctgcaaaagatcctcaacg
tgcaaaagaagctaccgatcatacaaaagatcatcatcatggatagcaagaccgactaccagggcttccaaagcatgtacaccttc
gtgacttcccatttgccacccggcttcaacgagtacgacttcgtgcccgagagcttcgaccgggacaaaaccatcgccctgatcatg
aacagtagtggcagtaccggattgcccaagggcgtagccctaccgcaccgcaccgcttgtgtccgattcagtcatgcccgcgacccc
atcttcggcaaccagatcatccccgacaccgctatcctcagcgtggtgccatttcaccacggcttcggcatgttcaccacgctgggct
acttgatctgcggctttcgggtcgtgctcatgtaccgcttcgaggaggagctattcttgcgcagcttgcaagactataagattcaatct
gccctgctggtgcccacactatttagcttcttcgctaagagcactctcatcgacaagtacgacctaagcaacttgcacgagatcgcca
gcggcggggcgccgctcagcaaggaggtaggtgaggccgtggccaaacgcttccacctaccaggcatccgccagggctacggcct
gacagaaacaaccagcgccattctgatcacccccgaaggggacgacaagcctggcgcagtaggcaaggtggtgcccttcttcgag
gctaaggtggtggacttggacaccggtaagacactgggtgtgaaccagcgcggcgagctgtgcgtccgtggccccatgatcatgag
cggctacgttaacaaccccgaggctacaaacgctctcatcgacaaggacggctggctgcacagcggcgacatcgcctactgggac
gaggacgagcacttcttcatcgtggaccggctgaagagcctgatcaaatacaagggctaccaggtagccccagccgaactggaga
gcatcctgctgcaacaccccaacatcttcgacgccggggtcgccggcctgcccgacgacgatgccggcgagctgcccgccgcagtc
gtcgtgctggaacacggtaaaaccatgaccgagaaggagatcgtggactatgtggccagccaggttacaaccgccaagaagctgc
gcggtggtgttgtgttcgtggacgaggtgcctaaaggactgaccggcaagttggacgcccgcaagatccgcgagattctcattaag
gccaagaagggcggcaagatcgccgtgtaataattctagagtcggggcggccggccgcttcgagcagacatgataagatacattg
atgagtttggacaaaccacaactagaatgcagtgaaaaaaatgctttatttgtgaaatttgtgatgctattgctttatttgtaaccatt
ataagctgcaataaacaagttaacaacaacaattgcattcattttatgtttcaggttcagggggaggtgtgggaggttttttaaagca
agtaaaacctctacaaatgtggtaaaatcgataaggatccgtcgaccgatgcccttgagagccttcaacccagtcagctccttccgg
tgggcgcggggcatgactatcgtcgccgcacttatgactgtcttctttatcatgcaactcgtaggacaggtgccggcagcgctcttcc
gcttcctcgctcactgactcgctgcgctcggtcgttcggctgcggcgagcggtatcagctcactcaaaggcggtaatacggttatcca
cagaatcaggggataacgcaggaaagaacatgtgagcaaaaggccagcaaaaggccaggaaccgtaaaaaggccgcgttgctg

gcgtttttccataggctccgcccccctgacgagcatcacaaaaatcgacgctcaagtcagaggtggcgaaacccgacaggactata
aagataccaggcgtttccccctggaagctccctcgtgcgctctcctgttccgaccctgccgcttaccggatacctgtccgcctttctccc
ttcgggaagcgtggcgctttctcatagctcacgctgtaggtatctcagttcggtgtaggtcgttcgctccaagctgggctgtgtgcacg
aaccccccgttcagcccgaccgctgcgccttatccggtaactatcgtcttgagtccaacccggtaagacacgacttatcgccactggc
agcagccactggtaacaggattagcagagcgaggtatgtaggcggtgctacagagttcttgaagtggtggcctaactacggctaca
ctagaagaacagtatttggtatctgcgctctgctgaagccagttaccttcggaaaaagagttggtagctcttgatccggcaaacaaa
ccaccgctggtagcggtggtttttttgtttgcaagcagcagattacgcgcagaaaaaaaggatctcaagaagatcctttgatcttttct
acggggtctgacgctcagtggaacgaaaactcacgttaagggattttggtcatgagattatcaaaaaggatcttcacctagatcctttt
taaattaaaaatgaagttttaaatcaatctaaagtatatatgagtaaacttggtctgacagcggccgcaaatgctaaaccactgcag
tggttaccagtgcttgatcagtgaggcaccgatctcagcgatctgcctatttcgttcgtccatagtggcctgactccccgtcgtgtaga
tcactacgattcgtgagggcttaccatcaggccccagcgcagcaatgatgccgcgagagccgcgttcaccggcccccgatttgtcag
caatgaaccagccagcagggagggccgagcgaagaagtggtcctgctactttgtccgcctccatccagtctatgagctgctgtcgtg
atgctagagtaagaagttcgccagtgagtagtttccgaagagttgtggccattgctactggcatcgtggtatcacgctcgtcgttcgg
tatggcttcgttcaactctggttcccagcggtcaagccgggtcacatgatcacccatattatgaagaaatgcagtcagctccttaggg
cctccgatcgttgtcagaagtaagttggccgcggtgttgtcgctcatggtaatggcagcactacacaattctcttaccgtcatgccatc
cgtaagatgcttttccgtgaccggcgagtactcaaccaagtcgttttgtgagtagtgtatacggcgaccaagctgctcttgcccggcg
tctatacgggacaacaccgcgccacatagcagtactttgaaagtgctcatcatcgggaatcgttcttcggggcggaaagactcaag
gatcttgccgctattgagatccagttcgatatagcccactcttgcacccagttgatcttcagcatcttttactttcaccagcgtttcggg
gtgtgcaaaaacaggcaagcaaaatgccgcaaagaagggaatgagtgcgacacgaaaatgttggatgctcatactcgtcctttttc
aatattattgaagcatttatcagggttactagtacgtctctcaaggataagtaagtaatattaaggtacgggaggtattggacaggcc
gcaataaaatatctttattttcattacatctgtgtgttggtttttgtgtgaatcgatagtactaacatacgctctccatcaaaacaaaa
cgaaacaaaacaaactagcaaaataggctgtccccagtgcaagtgcaggtgccagaacatttctct

ILPR 2G/C

GgcctaactggccggtacctGAGC**AAGCTTACAGGGGTGTGGGCACAGGGGTGTGGGCACAGGGGTGT**
CGCAGCGCAAAGAGCCCCGCCCTGCAGCCTCCAGCTCTCCTGGTCTAATGTGGAAAGTGGCCCAGG
TGAGGGCTTTGCTCTCCTGGAGACATTTGCCCCCAGCTGTGAGCAGGGACAGGTCTGGCCACCGGG
CCCCTGGTTAAGACTCTAATGACCCGCTGGTCCTGAGGAAGAGGTGCTGACGACCAAGGAGATCTT
CCCACAGACCCAGCACCAGGGAAATGGTCCGGAAATTGCAGCCTCAGCCCCCAGCCATCTGCCGAC
CCCCCCACCCCAGGCCCTAATGGGCCAGGCGGCAGGGGTTGAcAGGTAGGGGAGATGGGCTCTGA
GACTATAAAGCCAGCGGGGGCCCAGCAGCCCTCAGCCCTCCAGGACAGGCTGCATCAGAAGCTTggc
aatccggtactgttggtaaagccaccatggaagatgccaaaaacattaagaagggcccagcgccattctacccactcgaagacgg
gaccgccggcgagcagctgcacaaagccatgaagcgctacgccctggtgcccggcaccatcgcctttaccgacgcacatatcgag
gtggacattacctacgccgagtacttcgagatgagcgttcggctggcagaagctatgaagcgctatgggctgaatacaaaccatcg
gatcgtggtgtgcagcgagaatagcttgcagttcttcatgcccgtgttgggtgccctgttcatcggtgtggctgtggccccagctaacg
acatctacaacgagcgcgagctgctgaacagcatgggcatcagccagcccaccgtcgtattcgtgagcaagaaagggctgcaaaa
gatcctcaacgtgcaaaagaagctaccgatcatacaaaagatcatcatcatggatagcaagaccgactaccagggcttccaaagc
atgtacaccttcgtgacttcccatttgccacccggcttcaacgagtacgacttcgtgcccgagagcttcgaccgggacaaaaccatcg
ccctgatcatgaacagtagtggcagtaccggattgcccaagggcgtagccctaccgcaccgcaccgcttgtgtccgattcagtcatg
cccgcgaccccatcttcggcaaccagatcatccccgacaccgctatcctcagcgtggtgccatttcaccacggcttcggcatgttcac
cacgctgggctacttgatctgcggctttcgggtcgtgctcatgtaccgcttcgaggaggagctattcttgcgcagcttgcaagactata
agattcaatctgccctgctggtgcccacactatttagcttcttcgctaagagcactctcatcgacaagtacgacctaagcaacttgca
cgagatcgccagcggcggggcgccgctcagcaaggaggtaggtgaggccgtggccaaacgcttccacctaccaggcatccgccag
ggctacggcctgacagaaacaaccagcgccattctgatcacccccgaaggggacgacaagcctggcgcagtaggcaaggtggtgc
ccttcttcgaggctaaggtggtggacttggacaccggtaagacactgggtgtgaaccagcgcggcgagctgtgcgtccgtggcccc

atgatcatgagcggctacgttaacaaccccgaggctacaaacgctctcatcgacaaggacggctggctgcacagcggcgacatcg
cctactgggacgaggacgagcacttcttcatcgtggaccggctgaagagcctgatcaaatacaagggctaccaggtagccccagcc
gaactggagagcatcctgctgcaacaccccaacatcttcgacgccggggtcgccggcctgcccgacgacgatgccggcgagctgcc
cgccgcagtcgtcgtgctggaacacggtaaaaccatgaccgagaaggagatcgtggactatgtggccagccaggttacaaccgcc
aagaagctgcgcggtggtgttgtgttcgtggacgaggtgcctaaaggactgaccggcaagttggacgcccgcaagatccgcgagat
tctcattaaggccaagaagggcggcaagatcgccgtgtaataattctagagtcggggcggccggccgcttcgagcagacatgataa
gatacattgatgagtttggacaaaccacaactagaatgcagtgaaaaaaatgctttatttgtgaaatttgtgatgctattgctttattt
gtaaccattataagctgcaataaacaagttaacaacaacaattgcattcattttatgtttcaggttcagggggaggtgtgggaggttt
tttaaagcaagtaaaacctctacaaatgtggtaaaatcgataaggatccgtcgaccgatgcccttgagagccttcaacccagtcagc
tccttccggtgggcgcggggcatgactatcgtcgccgcacttatgactgtcttctttatcatgcaactcgtaggacaggtgccggcag
cgctcttccgcttcctcgctcactgactcgctgcgctcggtcgttcggctgcggcgagcggtatcagctcactcaaaggcggtaatac
ggttatccacagaatcaggggataacgcaggaaagaacatgtgagcaaaaggccagcaaaaggccaggaaccgtaaaaaggcc
gcgttgctggcgtttttccataggctccgcccccctgacgagcatcacaaaaatcgacgctcaagtcagaggtggcgaaacccgac
aggactataaagataccaggcgtttccccctggaagctccctcgtgcgctctcctgttccgaccctgccgcttaccggatacctgtccg
cctttctcccttcgggaagcgtggcgctttctcatagctcacgctgtaggtatctcagttcggtgtaggtcgttcgctccaagctgggct
gtgtgcacgaaccccccgttcagcccgaccgctgcgccttatccggtaactatcgtcttgagtccaacccggtaagacacgacttatc
gccactggcagcagccactggtaacaggattagcagagcgaggtatgtaggcggtgctacagagttcttgaagtggtggcctaact
acggctacactagaagaacagtatttggtatctgcgctctgctgaagccagttaccttcggaaaaagagttggtagctcttgatccgg
caaacaaaccaccgctggtagcggtggtttttttgtttgcaagcagcagattacgcgcagaaaaaaaggatctcaagaagatccttt
gatcttttctacggggtctgacgctcagtggaacgaaaactcacgttaagggattttggtcatgagattatcaaaaaggatcttcacc
tagatccttttaaattaaaaatgaagttttaaatcaatctaaagtatatatgagtaaacttggtctgacagcggccgcaaatgctaaa
ccactgcagtggttaccagtgcttgatcagtgaggcaccgatctcagcgatctgcctatttcgttcgtccatagtggcctgactccccg
tcgtgtagatcactacgattcgtgagggcttaccatcaggccccagcgcagcaatgatgccgcgagagccgcgttcaccggccccc
gatttgtcagcaatgaaccagccagcagggagggccgagcgaagaagtggtcctgctactttgtccgcctccatccagtctatgagc
tgctgtcgtgatgctagagtaagaagttcgccagtgagtagtttccgaagagttgtggccattgctactggcatcgtggtatcacgct
cgtcgttcggtatggcttcgttcaactctggttcccagcggtcaagccgggtcacatgatcacccatattatgaagaaatgcagtcag
ctccttagggcctccgatcgttgtcagaagtaagttggccgcggtgttgtcgctcatggtaatggcagcactacacaattctcttaccg
tcatgccatccgtaagatgcttttccgtgaccggcgagtactcaaccaagtcgttttgtgagtagtgtatacggcgaccaagctgctct
tgcccggcgtctatacgggacaacaccgcgccacatagcagtactttgaaagtgctcatcatcgggaatcgttcttcggggcggaaa
gactcaaggatcttgccgctattgagatccagttcgatatagcccactcttgcacccagttgatcttcagcatcttttactttcaccagc
gtttcggggtgtgcaaaaacaggcaagcaaaatgccgcaaagaagggaatgagtgcgacacgaaaatgttggatgctcatactcg
tcctttttcaatattattgaagcatttatcagggttactagtacgtctctcaaggataagtaagtaatattaaggtacgggaggtattg
gacaggccgcaataaaatatctttattttcattacatctgtgtgttggtttttttgtgtgaatcgatagtactaacatacgctctccatca
aaacaaaacgaaacaaaacaaactagcaaaataggctgtccccagtgcaagtgcaggtgccagaacatttctct

ILPR 4G/C

Ggcctaactggccggtacct GAGC**<u>GCGGCCGC</u>ATAGGGGTGTGGGGATAGGGGTGTGGGGATAGGGGT**
GTCGCAGCGCAAAGAGCCCCGCCCTGCAGCCTCCAGCTCTCCTGGTCTAATGTGGAAAGTGGCCCA
GGTGAGGGCTTTGCTCTCCTGGAGACATTTGCCCCCAGCTGTGAGCAGGGACAGGTCTGGCCACCG
GGCCCCTGGTTAAGACTCTAATGACCCGCTGGTCCTGAGGAAGAGGTGCTGACGACCAAGGAGATC
TTCCCACAGACCCAGCACCAGGGAAATGGTCCGGAAATTGCAGCCTCAGCCCCCAGCCATCTGCCGA
CCCCCCCACCCCAGGCCCTAATGGGCCAGGCGGCAGGGGTTGAcAGGTAGGGGAGATGGGCTCTG
AGACTATAAAGCCAGCGGGGGCCCAGCAGCCCTCAGCCCTCCAGGACAGGCTGCATCAGAAGCTTg
gcaatccggtactgttggtaaagccaccatggaagatgccaaaaacattaagaagggcccagcgccattctacccactcgaagac
gggaccgccggcgagcagctgcacaaagccatgaagcgctacgccctggtgcccggcaccatcgcctttaccgacgcacatatcg
aggtggacattacctacgccgagtacttcgagatgagcgttcggctggcagaagctatgaagcgctatgggctgaatacaaaccat

cggatcgtggtgtgcagcgagaatagcttgcagttcttcatgcccgtgttgggtgccctgttcatcggtgtggctgtggccccagctaa
cgacatctacaacgagcgcgagctgctgaacagcatgggcatcagccagcccaccgtcgtattcgtgagcaagaaagggctgcaa
aagatcctcaacgtgcaaaagaagctaccgatcatacaaaagatcatcatcatggatagcaagaccgactaccagggcttccaaa
gcatgtacaccttcgtgacttcccatttgccacccggcttcaacgagtacgacttcgtgcccgagagcttcgaccgggacaaaaccat
cgccctgatcatgaacagtagtggcagtaccggattgcccaagggcgtagccctaccgcaccgcaccgcttgtgtccgattcagtca
tgcccgcgaccccatcttcggcaaccagatcatccccgacaccgctatcctcagcgtggtgccatttcaccacggcttcggcatgttc
accacgctgggctacttgatctgcggctttcgggtcgtgctcatgtaccgcttcgaggaggagctattcttgcgcagcttgcaagact
ataagattcaatctgccctgctggtgcccacactatttagcttcttcgctaagagcactctcatcgacaagtacgacctaagcaacttg
cacgagatcgccagcggcggggcgccgctcagcaaggaggtaggtgaggccgtggccaaacgcttccacctaccaggcatccgcc
agggctacggcctgacagaaacaaccagcgccattctgatcacccccgaaggggacgacaagcctggcgcagtaggcaaggtgg
tgcccttcttcgaggctaaggtggtggacttggacaccggtaagacactgggtgtgaaccagcgcggcgagctgtgcgtccgtggcc
ccatgatcatgagcggctacgttaacaaccccgaggctacaaacgctctcatcgacaaggacggctggctgcacagcggcgacatc
gcctactgggacgaggacgagcacttcttcatcgtggaccggctgaagagcctgatcaaatacaagggctaccaggtagccccagc
cgaactggagagcatcctgctgcaacaccccaacatcttcgacgccggggtcgccggcctgcccgacgacgatgccggcgagctgc
ccgccgcagtcgtcgtgctggaacacggtaaaaccatgaccgagaaggagatcgtggactatgtggccagccaggttacaaccgc
caagaagctgcgcggtggtgttgtgttcgtggacgaggtgcctaaaggactgaccggcaagttggacgcccgcaagatccgcgag
attctcattaaggccaagaagggcggcaagatcgccgtgtaataattctagagtcggggcggccggccgcttcgagcagacatgat
aagatacattgatgagtttggacaaaccacaactagaatgcagtgaaaaaaatgctttatttgtgaaatttgtgatgctattgctttat
ttgtaaccattataagctgcaataaacaagttaacaacaacaattgcattcattttatgtttcaggttcagggggaggtgtgggaggt
tttttaaagcaagtaaaacctctacaaatgtggtaaaatcgataaggatccgtcgaccgatgcccttgagagccttcaacccagtca
gctccttccggtgggcgcggggcatgactatcgtcgccgcacttatgactgtcttctttatcatgcaactcgtaggacaggtgccggc
agcgctcttccgcttcctcgctcactgactcgctgcgctcggtcgttcggctgcggcgagcggtatcagctcactcaaaggcggtaat
acggttatccacagaatcaggggataacgcaggaaagaacatgtgagcaaaaggccagcaaaaggccaggaaccgtaaaaagg
ccgcgttgctggcgtttttccataggctccgcccccctgacgagcatcacaaaaatcgacgctcaagtcagaggtggcgaaacccga
caggactataaagataccaggcgtttccccctggaagctccctcgtgcgctctcctgttccgaccctgccgcttaccggatacctgtcc
gcctttctcccttcgggaagcgtggcgctttctcatagctcacgctgtaggtatctcagttcggtgtaggtcgttcgctccaagctgggc
tgtgtgcacgaaccccccgttcagcccgaccgctgcgccttatccggtaactatcgtcttgagtccaacccggtaagacacgacttat
cgccactggcagcagccactggtaacaggattagcagagcgaggtatgtaggcggtgctacagagttcttgaagtggtggcctaac
tacggctacactagaagaacagtatttggtatctgcgctctgctgaagccagttaccttcggaaaaagagttggtagctcttgatccg
gcaaacaaaccaccgctggtagcggtggtttttttgtttgcaagcagcagattacgcgcagaaaaaaaggatctcaagaagatcctt
tgatcttttctacggggtctgacgctcagtggaacgaaaactcacgttaagggattttggtcatgagattatcaaaaaggatcttcac
ctagatccttttaaattaaaaatgaagttttaaatcaatctaaagtatatatgagtaaacttggtctgacagcggccgcaaatgctaa
accactgcagtggttaccagtgcttgatcagtgaggcaccgatctcagcgatctgcctatttcgttcgtccatagtggcctgactcccc
gtcgtgtagatcactacgattcgtgagggcttaccatcaggccccagcgcagcaatgatgccgcgagagccgcgttcaccggcccc
cgatttgtcagcaatgaaccagccagcagggagggccgagcgaagaagtggtcctgctactttgtccgcctccatccagtctatgag
ctgctgtcgtgatgctagagtaagaagttcgccagtgagtagtttccgaagagttgtggccattgctactggcatcgtggtatcacgc
tcgtcgttcggtatggcttcgttcaactctggttcccagcggtcaagccgggtcacatgatcacccatattatgaagaaatgcagtca
gctccttagggcctccgatcgttgtcagaagtaagttggccgcggtgttgtcgctcatggtaatggcagcactacacaattctcttacc
gtcatgccatccgtaagatgcttttccgtgaccggcgagtactcaaccaagtcgttttgtgagtagtgtatacggcgaccaagctgct
cttgcccggcgtctatacgggacaacaccgcgccacatagcagtactttgaaagtgctcatcatcgggaatcgttcttcggggcgga
aagactcaaggatcttgccgctattgagatccagttcgatatagcccactcttgcacccagttgatcttcagcatcttttactttcacca
gcgtttcggggtgtgcaaaaacaggcaagcaaaatgccgcaaagaagggaatgagtgcgacacgaaaatgttggatgctcatact
cgtcctttttcaatattattgaagcatttatcagggttactagtacgtctctcaaggataagtaagtaatattaaggtacgggaggtat
tggacaggccgcaataaaatatctttattttcattacatctgtgtgttggtttttttgtgtgaatcgatagtactaacatacgctctccatc
aaaacaaaacgaaacaaaacaaactagcaaaataggctgtccccagtgcaagtgcaggtgccagaacatttctct

ILPR 10G/C

Ggcctaactggccggtacct GAGC**CCATGGACAGGGTCCTGGGGACAGGGTCCTGGGGACAGGGTCCT**C
GCAGCGCAAAGAGCCCCGCCCTGCAGCCTCCAGCTCTCCTGGTCTAATGTGGAAAGTGGCCCAGGT
GAGGGCTTTGCTCTCCTGGAGACATTTGCCCCCAGCTGTGAGCAGGGACAGGTCTGGCCACCGGGC
CCCTGGTTAAGACTCTAATGACCCGCTGGTCCTGAGGAAGAGGTGCTGACGACCAAGGAGATCTTC
CCACAGACCCAGCACCAGGGAAATGGTCCGGAAATTGCAGCCTCAGCCCCCAGCCATCTGCCGACC
CCCCCACCCCAGGCCCTAATGGGCCAGGCGGCAGGGGTTGAcAGGTAGGGGAGATGGGCTCTGAG
ACTATAAAGCCAGCGGGGGCCCAGCAGCCCTCAGCCCTCCAGGACAGGCTGCATCAGAAGCTTggca
atccggtactgttggtaaagccaccatggaagatgccaaaaacattaagaagggcccagcgccattctacccactcgaagacggg
accgccggcgagcagctgcacaaagccatgaagcgctacgccctggtgcccggcaccatcgcctttaccgacgcacatatcgaggt
ggacattacctacgccgagtacttcgagatgagcgttcggctggcagaagctatgaagcgctatgggctgaatacaaaccatcgga
tcgtggtgtgcagcgagaatagcttgcagttcttcatgcccgtgttgggtgccctgttcatcggtgtggctgtggccccagctaacgac
atctacaacgagcgcgagctgctgaacagcatgggcatcagccagcccaccgtcgtattcgtgagcaagaaagggctgcaaaaga
tcctcaacgtgcaaaagaagctaccgatcatacaaaagatcatcatcatggatagcaagaccgactaccagggcttccaaagcat
gtacaccttcgtgacttcccatttgccacccggcttcaacgagtacgacttcgtgcccgagagcttcgaccgggacaaaaccatcgcc
ctgatcatgaacagtagtggcagtaccggattgcccaagggcgtagccctaccgcaccgcaccgcttgtgtccgattcagtcatgcc
cgcgaccccatcttcggcaaccagatcatccccgacaccgctatcctcagcgtggtgccatttcaccacggcttcggcatgttcacca
cgctgggctacttgatctgcggctttcgggtcgtgctcatgtaccgcttcgaggaggagctattcttgcgcagcttgcaagactataag
attcaatctgccctgctggtgcccacactatttagcttcttcgctaagagcactctcatcgacaagtacgacctaagcaacttgcacg
agatcgccagcggcggggcgccgctcagcaaggaggtaggtgaggccgtggccaaacgcttccacctaccaggcatccgccaggg
ctacggcctgacagaaacaaccagcgccattctgatcacccccgaaggggacgacaagcctggcgcagtaggcaaggtggtgccc
ttcttcgaggctaaggtggtggacttggacaccggtaagacactgggtgtgaaccagcgcggcgagctgtgcgtccgtggccccatg
atcatgagcggctacgttaacaaccccgaggctacaaacgctctcatcgacaaggacggctggctgcacagcggcgacatcgccta
ctgggacgaggacgagcacttcttcatcgtggaccggctgaagagcctgatcaaatacaagggctaccaggtagccccagccgaa
ctggagagcatcctgctgcaacaccccaacatcttcgacgccggggtcgccggcctgcccgacgacgatgccggcgagctgcccgc
cgcagtcgtcgtgctggaacacggtaaaaccatgaccgagaaggagatcgtggactatgtggccagccaggttacaaccgccaag
aagctgcgcggtggtgttgtgttcgtggacgaggtgcctaaaggactgaccggcaagttggacgcccgcaagatccgcgagattct
cattaaggccaagaagggcggcaagatcgccgtgtaataattctagagtcggggcggccggccgcttcgagcagacatgataaga
tacattgatgagtttggacaaaccacaactagaatgcagtgaaaaaaatgctttatttgtgaaatttgtgatgctattgctttatttgt
aaccattataagctgcaataaacaagttaacaacaacaattgcattcattttatgtttcaggttcagggggaggtgtgggaggttttt
taaagcaagtaaaacctctacaaatgtggtaaaatcgataaggatccgtcgaccgatgcccttgagagccttcaacccagtcagct
ccttccggtgggcgcggggcatgactatcgtcgccgcacttatgactgtcttctttatcatgcaactcgtaggacaggtgccggcagc
gctcttccgcttcctcgctcactgactcgctgcgctcggtcgttcggctgcggcgagcggtatcagctcactcaaaggcggtaatacg
gttatccacagaatcaggggataacgcaggaaagaacatgtgagcaaaaggccagcaaaaggccaggaaccgtaaaaaggccg
cgttgctggcgtttttccataggctccgcccccctgacgagcatcacaaaaatcgacgctcaagtcagaggtggcgaaacccgaca
ggactataaagataccaggcgtttccccctggaagctccctcgtgcgctctcctgttccgaccctgccgcttaccggatacctgtccgc
ctttctcccttcgggaagcgtggcgctttctcatagctcacgctgtaggtatctcagttcggtgtaggtcgttcgctccaagctgggctg
tgtgcacgaaccccccgttcagcccgaccgctgcgccttatccggtaactatcgtcttgagtccaacccggtaagacacgacttatcg
ccactggcagcagccactggtaacaggattagcagagcgaggtatgtaggcggtgctacagagttcttgaagtggtggcctaacta
cggctacactagaagaacagtatttggtatctgcgctctgctgaagccagttaccttcggaaaaagagttggtagctcttgatccggc
aaacaaaccaccgctggtagcggtggtttttttgtttgcaagcagcagattacgcgcagaaaaaaaggatctcaagaagatcctttg
atcttttctacggggtctgacgctcagtggaacgaaaactcacgttaagggattttggtcatgagattatcaaaaaggatcttcacct
agatccttttaaattaaaaatgaagttttaaatcaatctaaagtatatatgagtaaacttggtctgacagcggccgcaaatgctaaac
cactgcagtggttaccagtgcttgatcagtgaggcaccgatctcagcgatctgcctatttcgttcgtccatagtggcctgactccccgt
cgtgtagatcactacgattcgtgagggcttaccatcaggccccagcgcagcaatgatgccgcgagagccgcgttcaccggcccccg

atttgtcagcaatgaaccagccagcagggagggccgagcgaagaagtggtcctgctactttgtccgcctccatccagtctatgagct
gctgtcgtgatgctagagtaagaagttcgccagtgagtagtttccgaagagttgtggccattgctactggcatcgtggtatcacgctc
gtcgttcggtatggcttcgttcaactctggttcccagcggtcaagccgggtcacatgatcacccatattatgaagaaatgcagtcagc
tccttagggcctccgatcgttgtcagaagtaagttggccgcggtgttgtcgctcatggtaatggcagcactacacaattctcttaccgt
catgccatccgtaagatgcttttccgtgaccggcgagtactcaaccaagtcgttttgtgagtagtgtatacggcgaccaagctgctctt
gcccggcgtctatacgggacaacaccgcgccacatagcagtactttgaaagtgctcatcatcgggaatcgttcttcggggcggaaa
gactcaaggatcttgccgctattgagatccagttcgatatagcccactcttgcacccagttgatcttcagcatcttttactttcaccagc
gtttcggggtgtgcaaaaacaggcaagcaaaatgccgcaaagaagggaatgagtgcgacacgaaaatgttggatgctcatactcg
tcctttttcaatattattgaagcatttatcagggttactagtacgtctctcaaggataagtaagtaatattaaggtacgggaggtattg
gacaggccgcaataaaatatctttattttcattacatctgtgtgttggtttttttgtgtgaatcgatagtactaacatacgctctccatca
aaacaaaacgaaacaaaacaaactagcaaaataggctgtccccagtgcaagtgcaggtgccagaacatttctct

3) SUPPLEMENTARY FIGURES

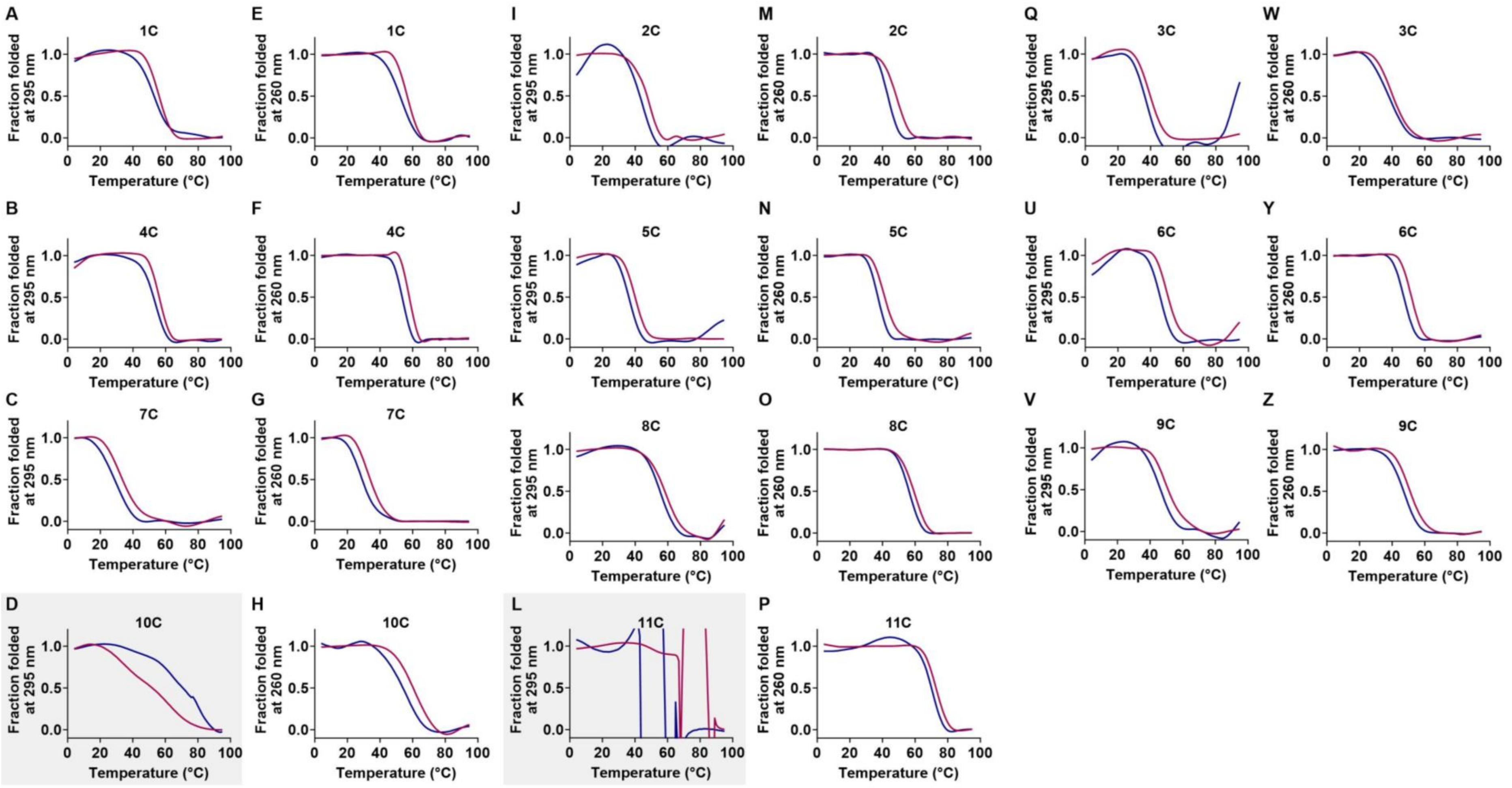

Supplementary Figure 1. Fraction folded UV **melting** (purple) and annealing (blue) of C-rich ILPR sequences showing representative spectra at 295 nm (A-D, I-L, Q-V) and 260 nm (E-H, M-P, W-Z) of 2.5 µM DNA in 10 mM NaCaco 100 mM KCl at pH 5.5. Graphs D and L (in grey) showed no repeatable thermodynamic profile at 295 nm. Source data for this figure are provided as a Source Data file.

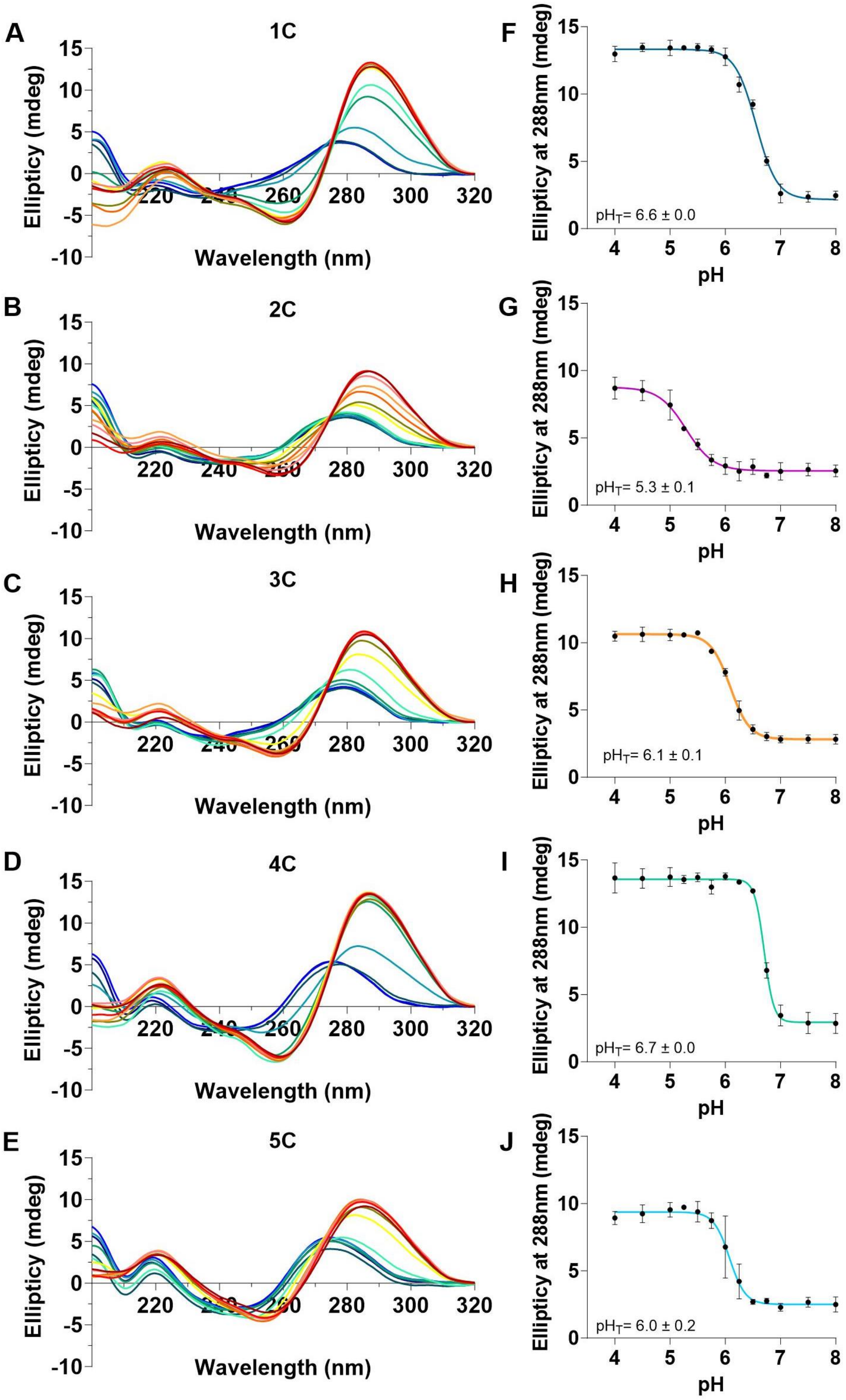

Supplementary Figure 2. A-E) CD spectroscopy of C-rich ILPR sequences 1C, 2C, 3C, 4C, 5C. 10 µM DNA in 10 mM NaCaco 100 mM KCl and pH as indicated. F-J) Corresponding plot ellipticity of the experimental repeats (n=3, mean ± SD) at 288 nm at the different pHs to determine

transitional pH (pH_T) from the inflection point of the Boltzmann sigmoidal. Source data for this figure are provided as a Source Data file.

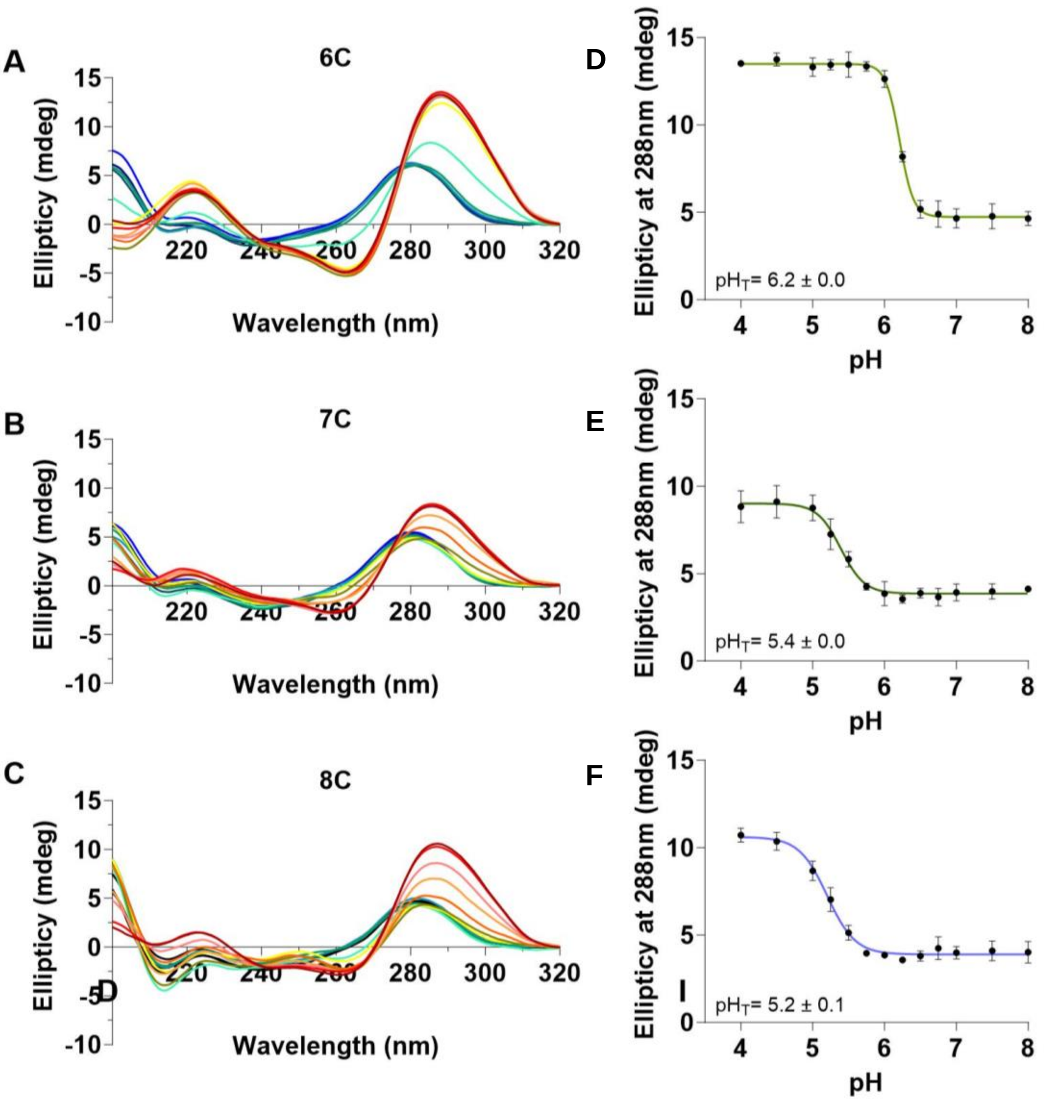

Supplementary Figure 3. A-C) CD spectroscopy of C-rich ILPR sequences 6C, 7C, 8C. 10 µM DNA in 10 mM NaCaco 100 mM KCl and pH as indicated. D-F) Corresponding plot ellipticity of the experimental repeats (n=3, mean ± SD) at 288 nm at the different pHs to determine transitional pH (pH_T) from the inflection point of the Boltzmann sigmoidal. Source data for this figure are provided as a Source Data file.

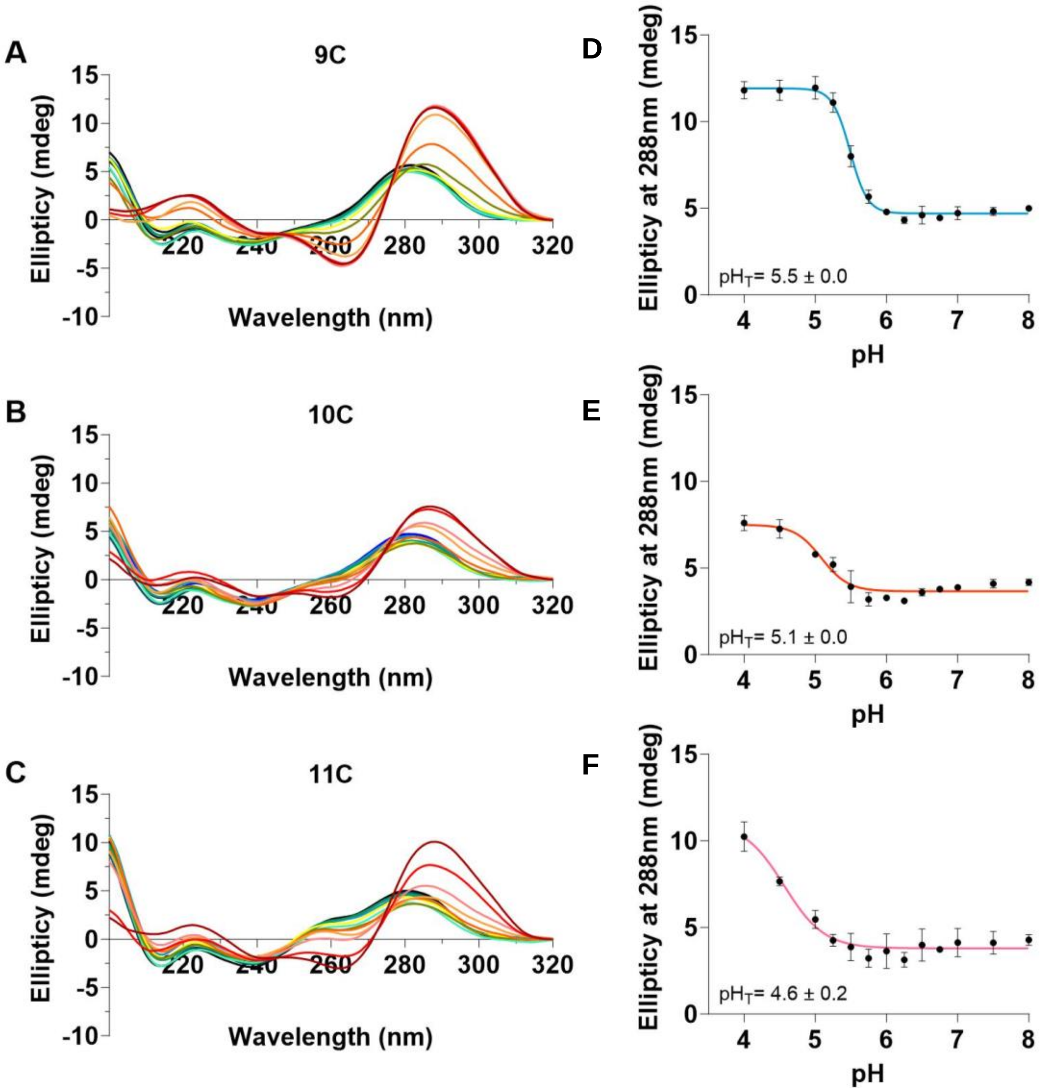

Supplementary Figure 4. A-C) CD spectroscopy of C-rich ILPR sequences 9C, 10C, 11C. 10 µM DNA in 10 mM NaCaco 100 mM KCl and pH as indicated. D-F) Corresponding plot ellipticity of the experimental repeats (n=3, mean ± SD) at 288 nm at the different pHs to determine transitional pH (pH_T) from the inflection point of the Boltzmann sigmoidal. Source data for this figure are provided as a Source Data file.

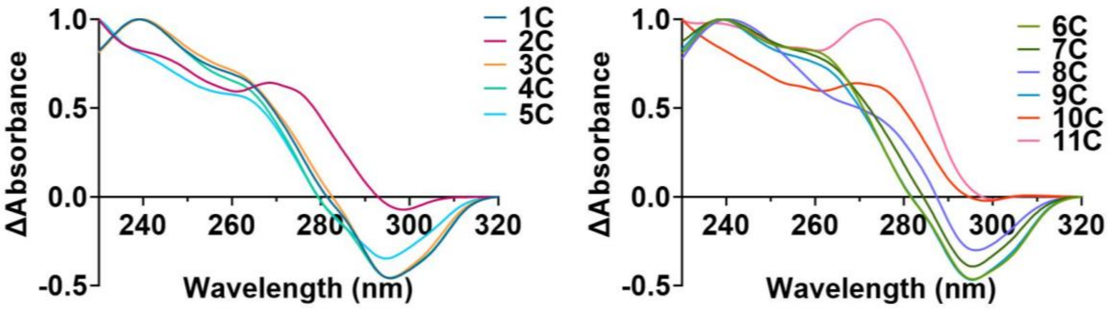

Supplementary Figure 5. Thermal difference spectra of C-rich ILPR sequences with 2.5 µM DNA in 10 mM NaCaco 100 mM KCl at pH 5.5. Source data for this figure are provided as a Source Data file.

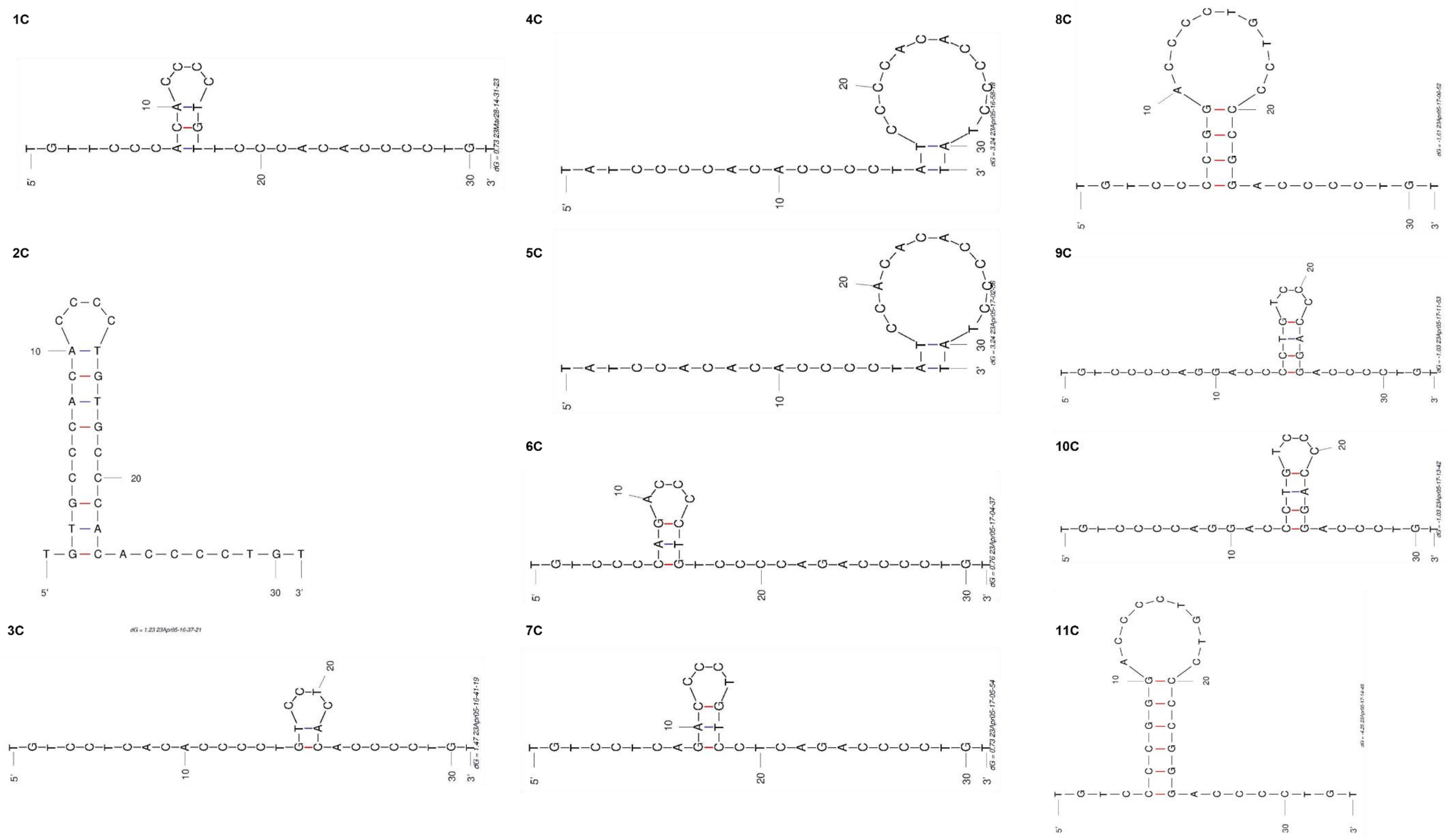

Supplementary Figure 6: M-fold predictions of secondary DNA structures with highest predicted thermal stability from C-rich ILPR sequences.[7]

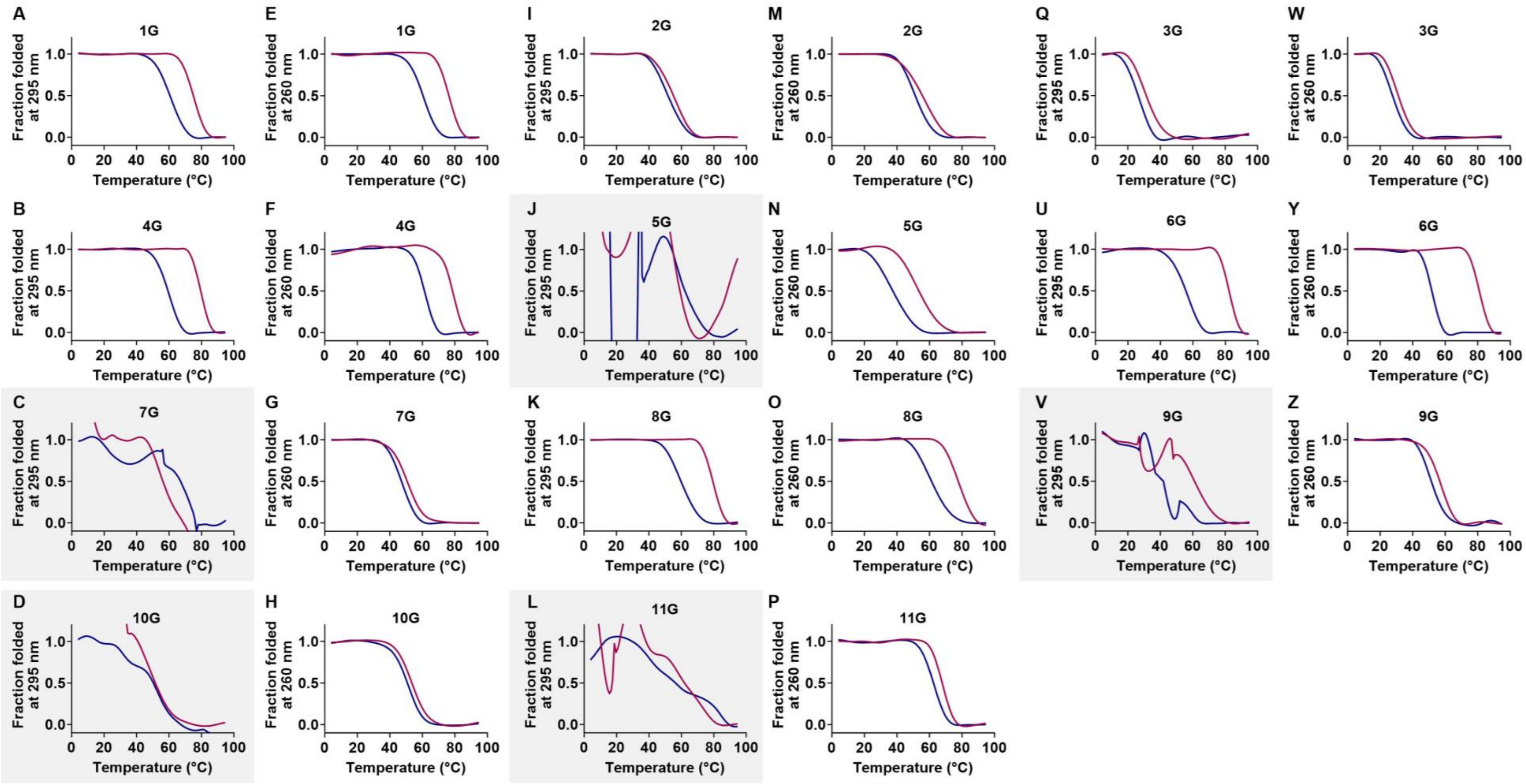

Supplementary Figure 7. Fraction folded UV melting (purple) and annealing (blue) of G-rich ILPR sequences showing representative spectra at 295 nm (A-D, I-L, Q-V) and 260 nm (E-H, M-P, W-Z) of 2.5 µM DNA in 10 mM NaCaco 20 mM KCl buffer at pH 7.0. Graphs D, J, L, and V (in grey) showed no repeatable thermodynamic profiles at 295 nm. Source data for this figure are provided as a Source Data file.

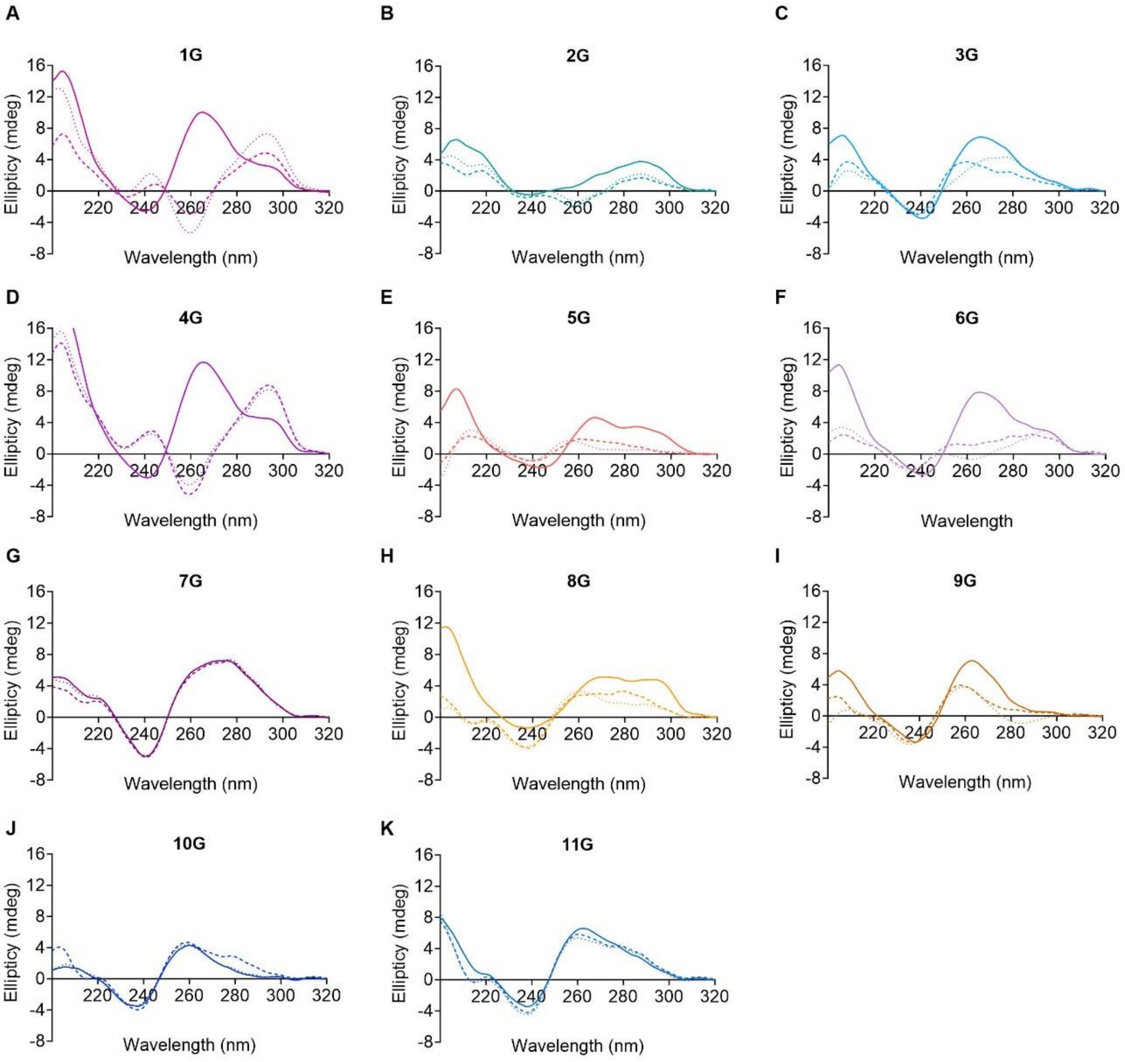

Supplementary Figure 8. Biophysical characterisation of G-rich ILPR sequences via CD spectroscopy using 10 µM DNA in 10 mM NaCaco 100 mM KCl at pH 7.0 (solid line), 10 mM NaCaco 100 mM LiCl at pH 7.0 (dashed lined), or 10 mM NaCaco 100 mM NaCl at pH 7.0 (dotted line). Panels A-K represent G-rich ILPR sequences 1G-11G as indicated. Source data for this figure are provided as a Source Data file.

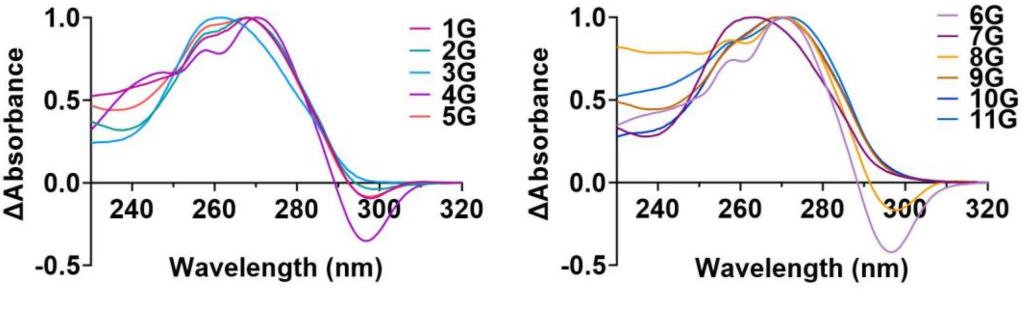

Supplementary Figure 9. Thermal difference spectra of G-rich ILPR sequences with 2.5 µM DNA in 10 mM NaCaco 20 mM KCl at pH 7.0. Source data for this figure are provided as a Source Data file.

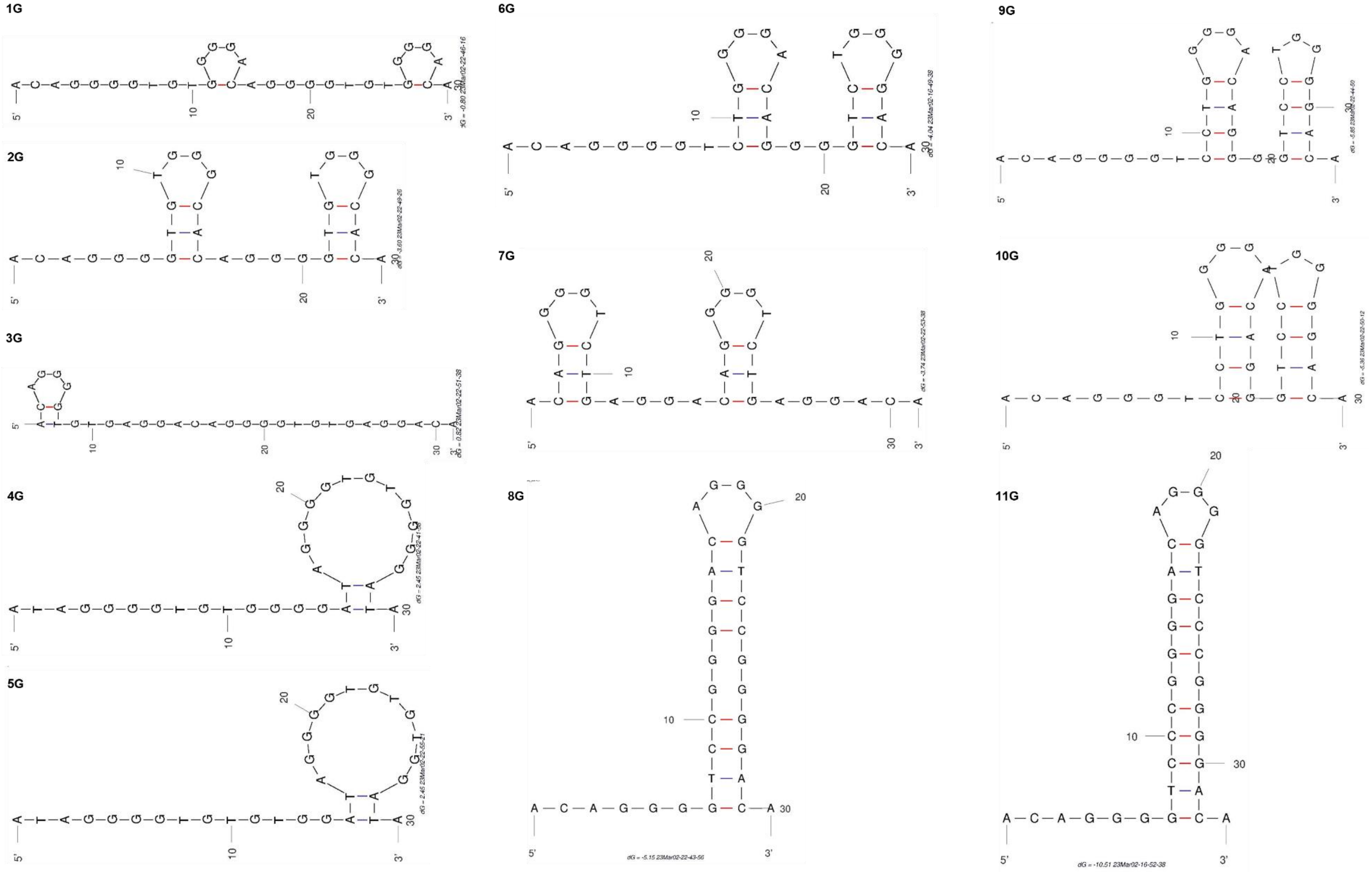

Supplementary Figure 10: M-fold predictions of secondary DNA structures with highest thermal stability of G-rich ILPR sequences.

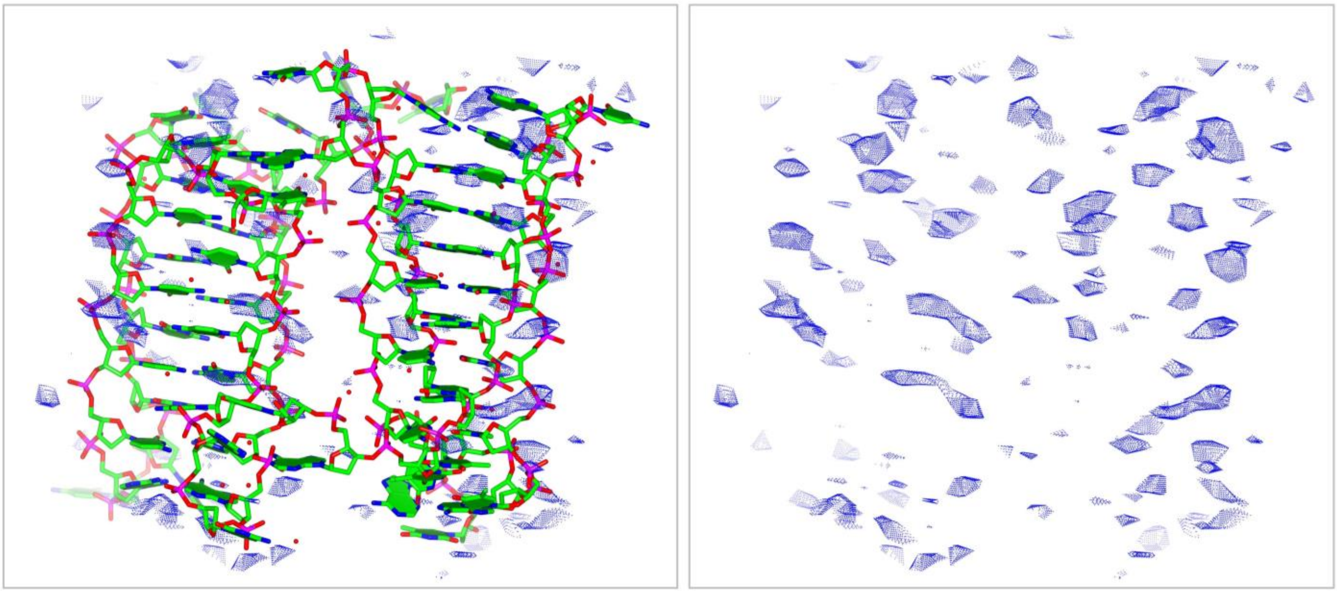

Supplementary Figure 11. Anomalous difference map ($F_{anom(calc)}$, blue, 2σ) showing P atoms positions, 3.1 keV data (λ = 3.9995 Å).

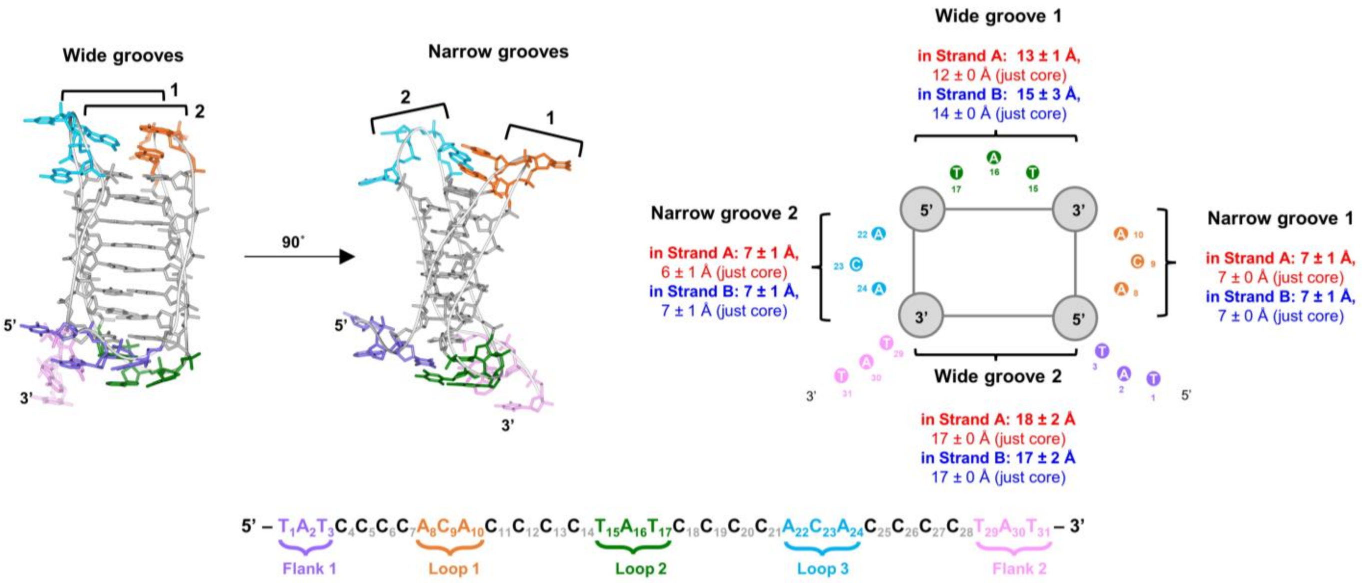

Supplementary Figure 12. Schematic of the grooves in the i-motif structure. Average dimensions for each groove are shown. The average dimension of the P-P distances at the core only was also calculated and included.

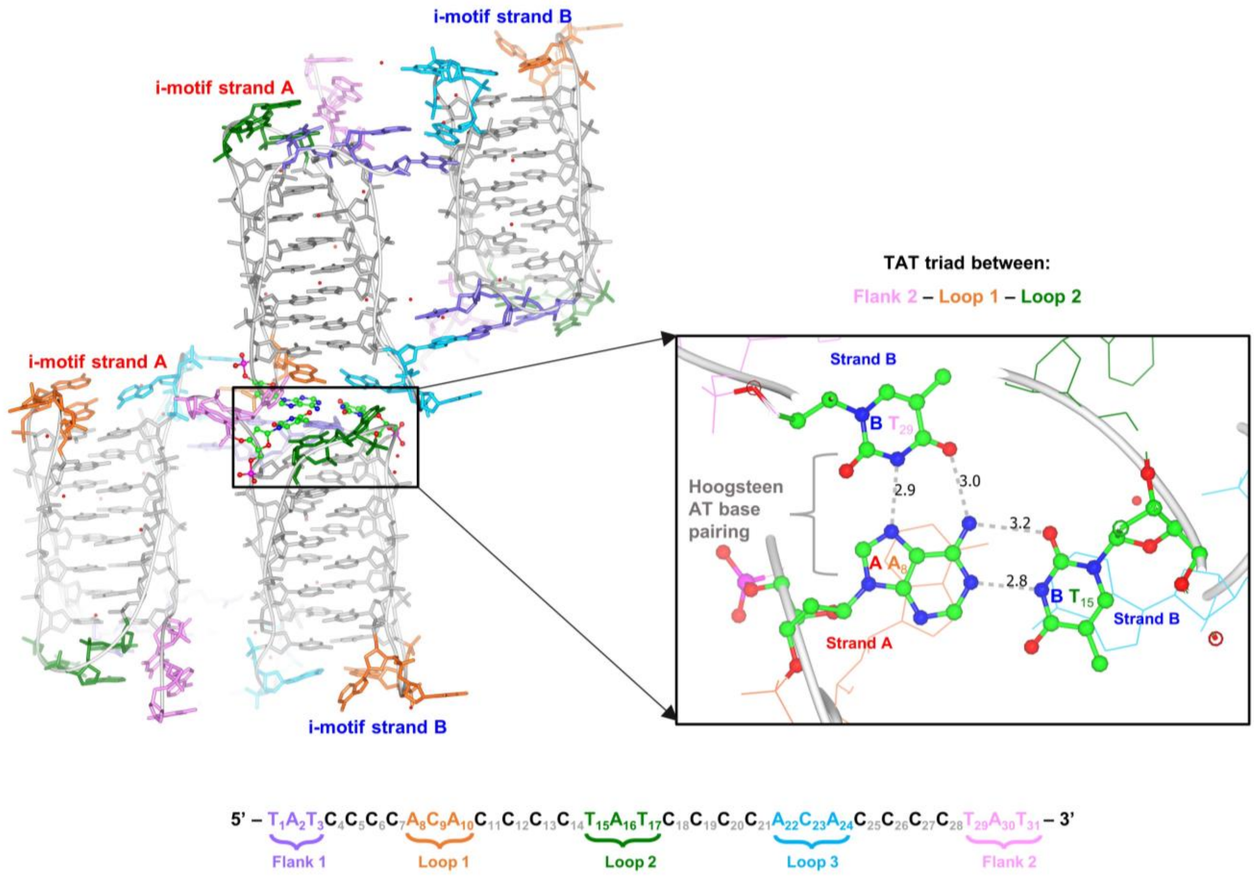

Supplementary Figure 13. Intramolecular 4C i-motifs A and B forming a TAT triad.

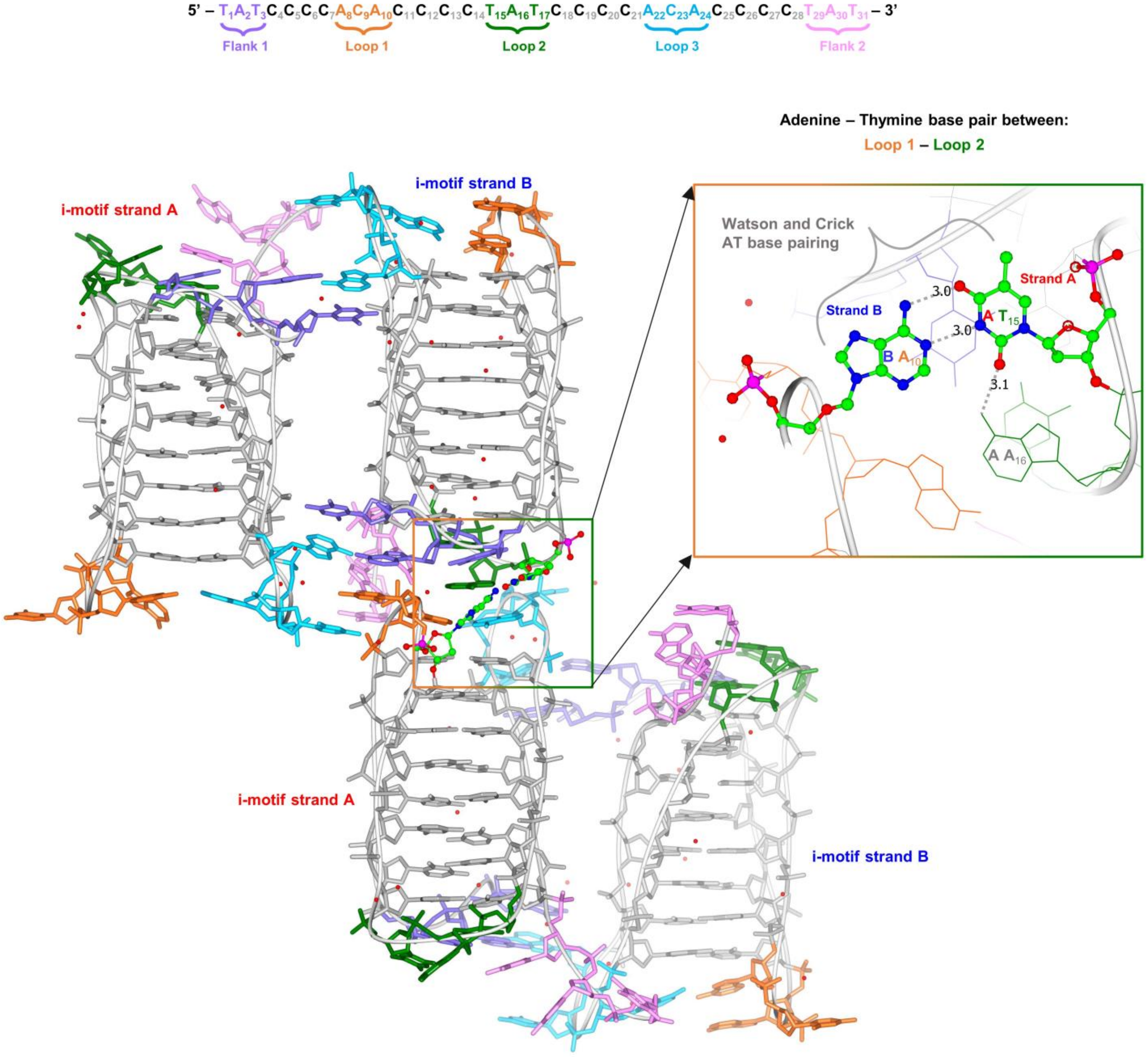

Supplementary Figure 14. Intramolecular 4C i-motifs A and B forming AT base pairs.

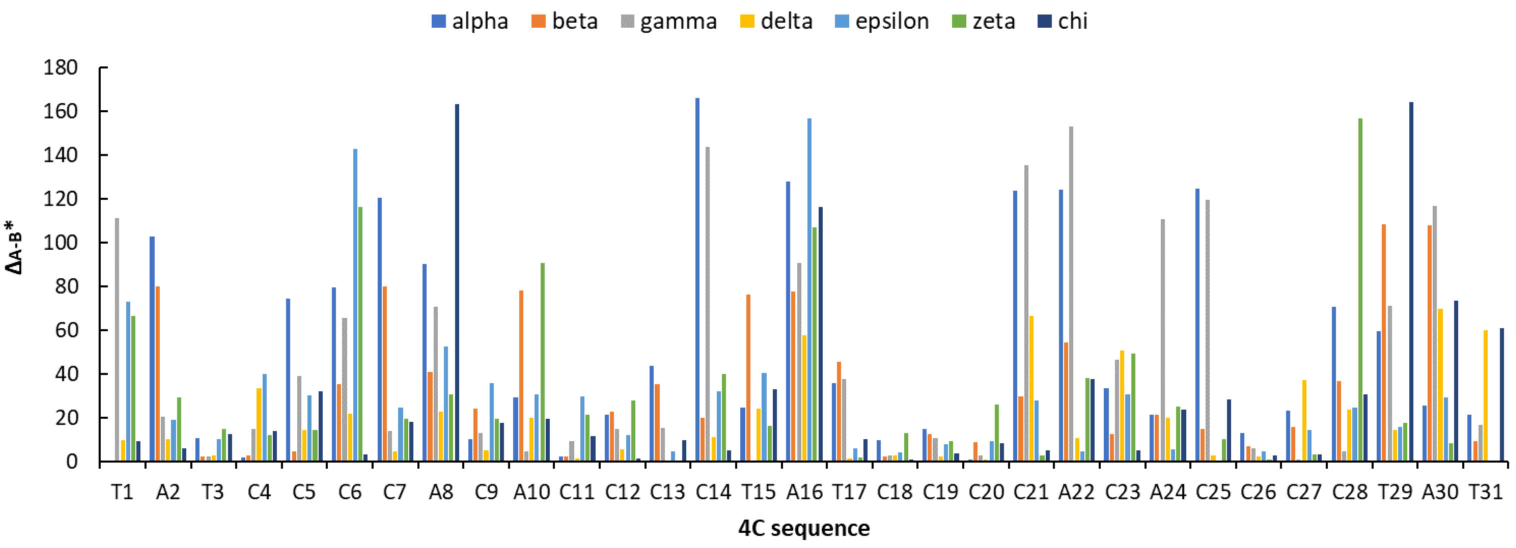

Supplementary Figure 15. Comparison of the torsion angles and sugar pucker between strand A and strand B. The smallest absolute difference between strand A and B within the range of 0° to 180° ($\Delta_{A\text{-}B}$*) is shown for each nucleotide of the 4C sequence.

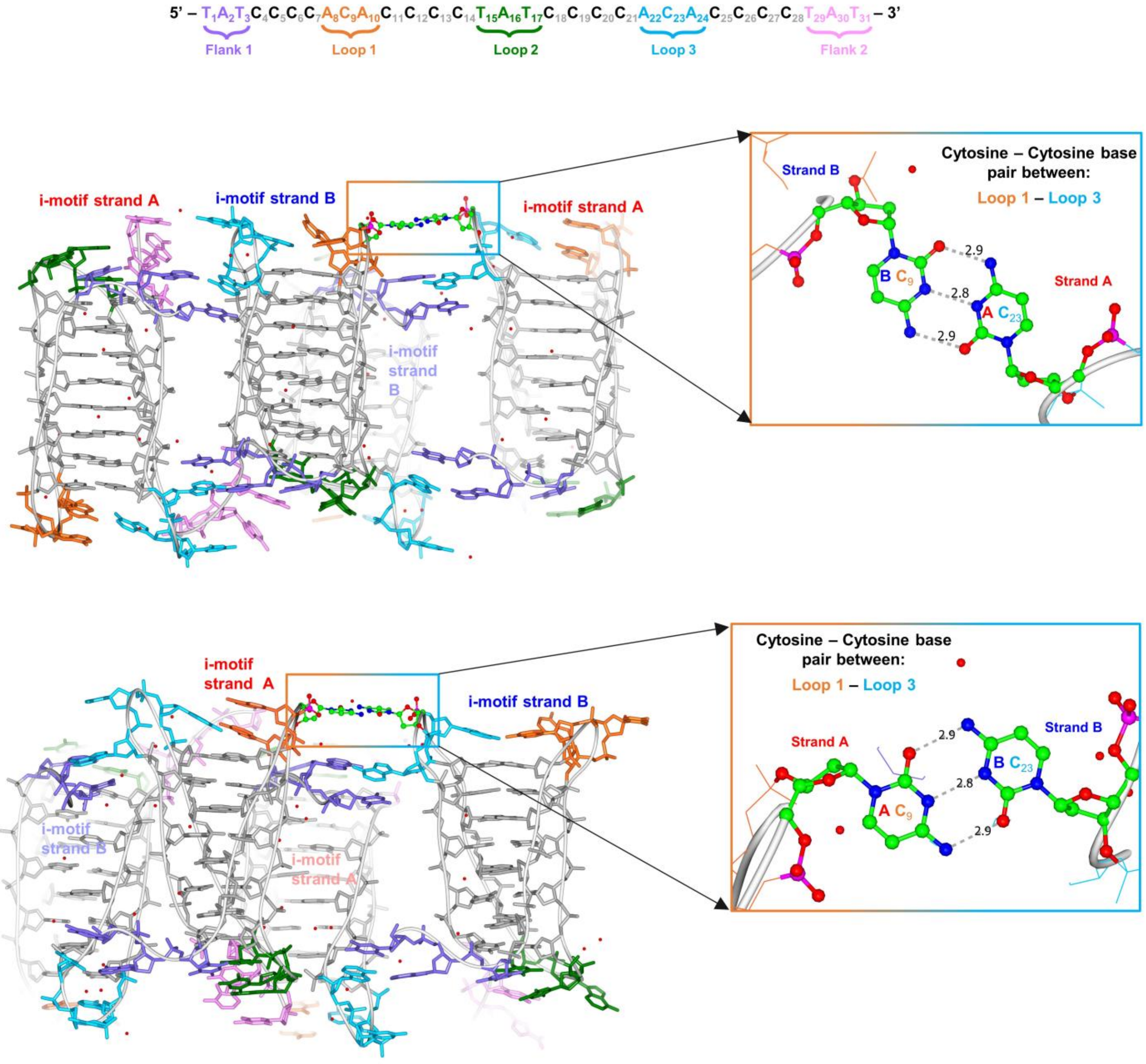

Supplementary Figure 16. Intramolecular 4C i-motifs A and B forming CC base pairs.

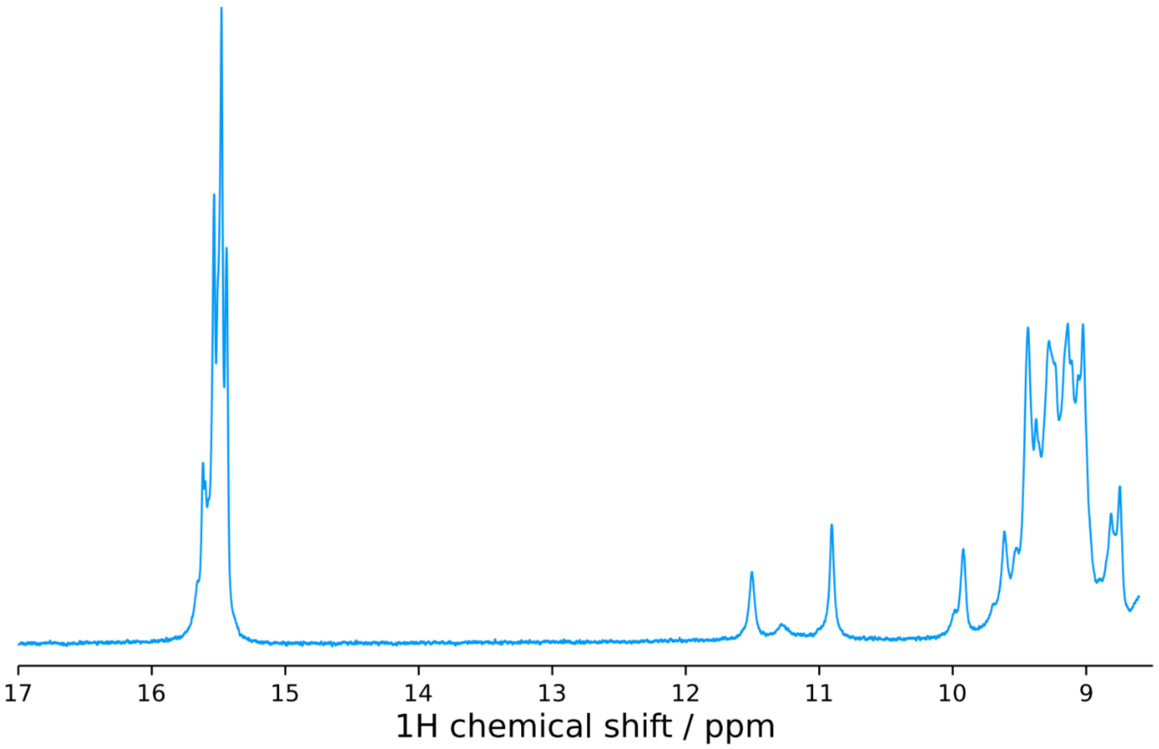

Supplementary Figure 17: ^{1}H-1D NMR of 4C 0.7 mM, 9.1 mM NaCaco, pH 5.5, 91 mM KCl, 17% D_2O at 298K.

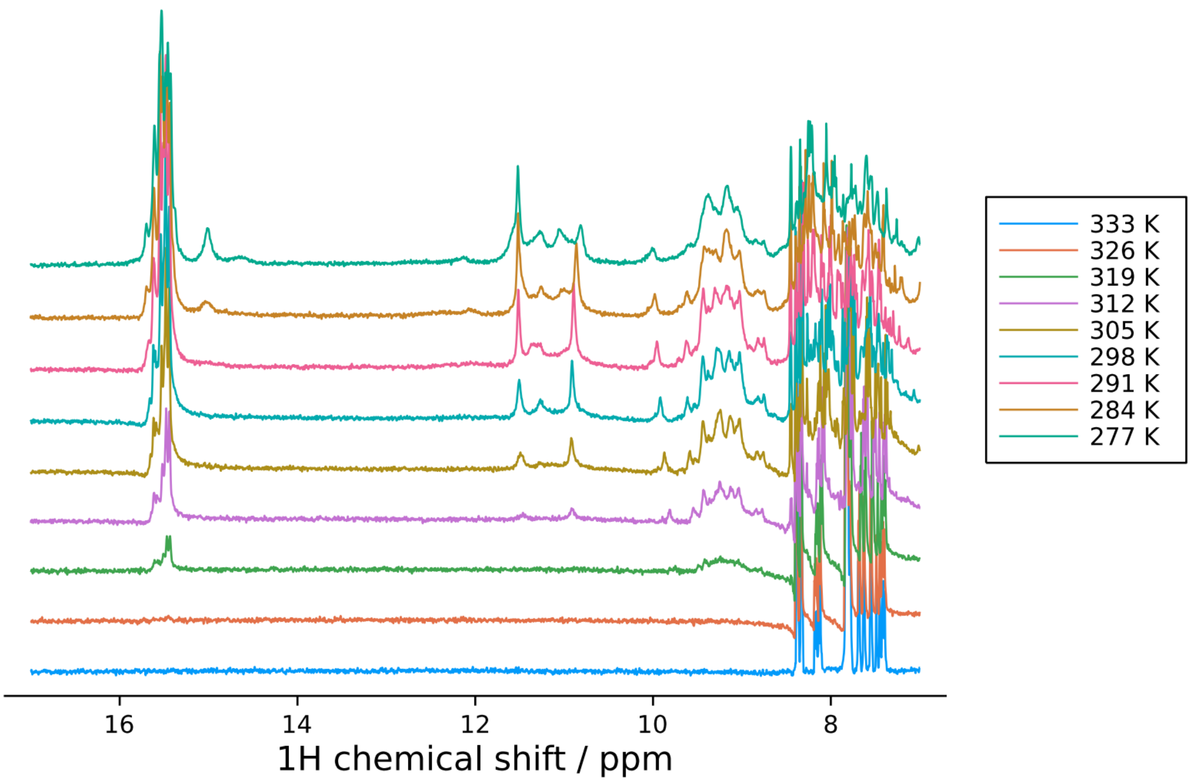

Supplementary Figure 18: Annealing ^{1}H-1D NMR of 4C 0.7 mM, 9.1 mM NaCaco, pH 5.5, 91 mM KCl, 17% D_2O between 333 and 277 K.

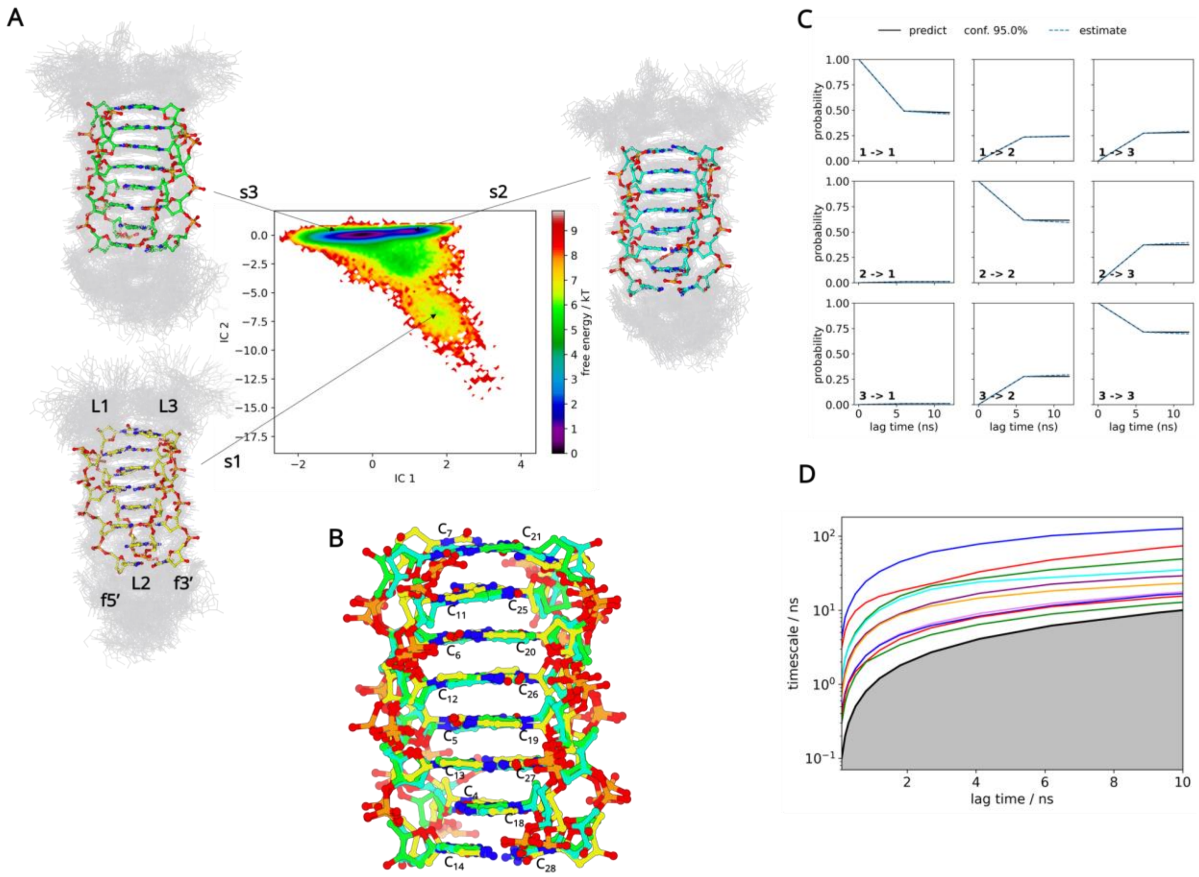

Supplementary Figure 19: (A) Projection of the Markov state model free energy landscape along the time-lagged independent components IC1 and IC2 of 4Cdel. The representative structures s1, s2 and s3 derived from the dynamics of the central stem are marked in their basins. The positions of the loops have been marked L1, L2 and L3, while that of the 5' and 3' flanking nucleotides are labelled f5' and f3' respectively. (B) Superimposition of the three representative central stem structures of 4Cdel. (C) A Chapman-Kolmogorov (CK) plot is used to validate the self-consistency of the built MSM. 3 macrostates were defined. (D) The implied timescale (ITS) plot.

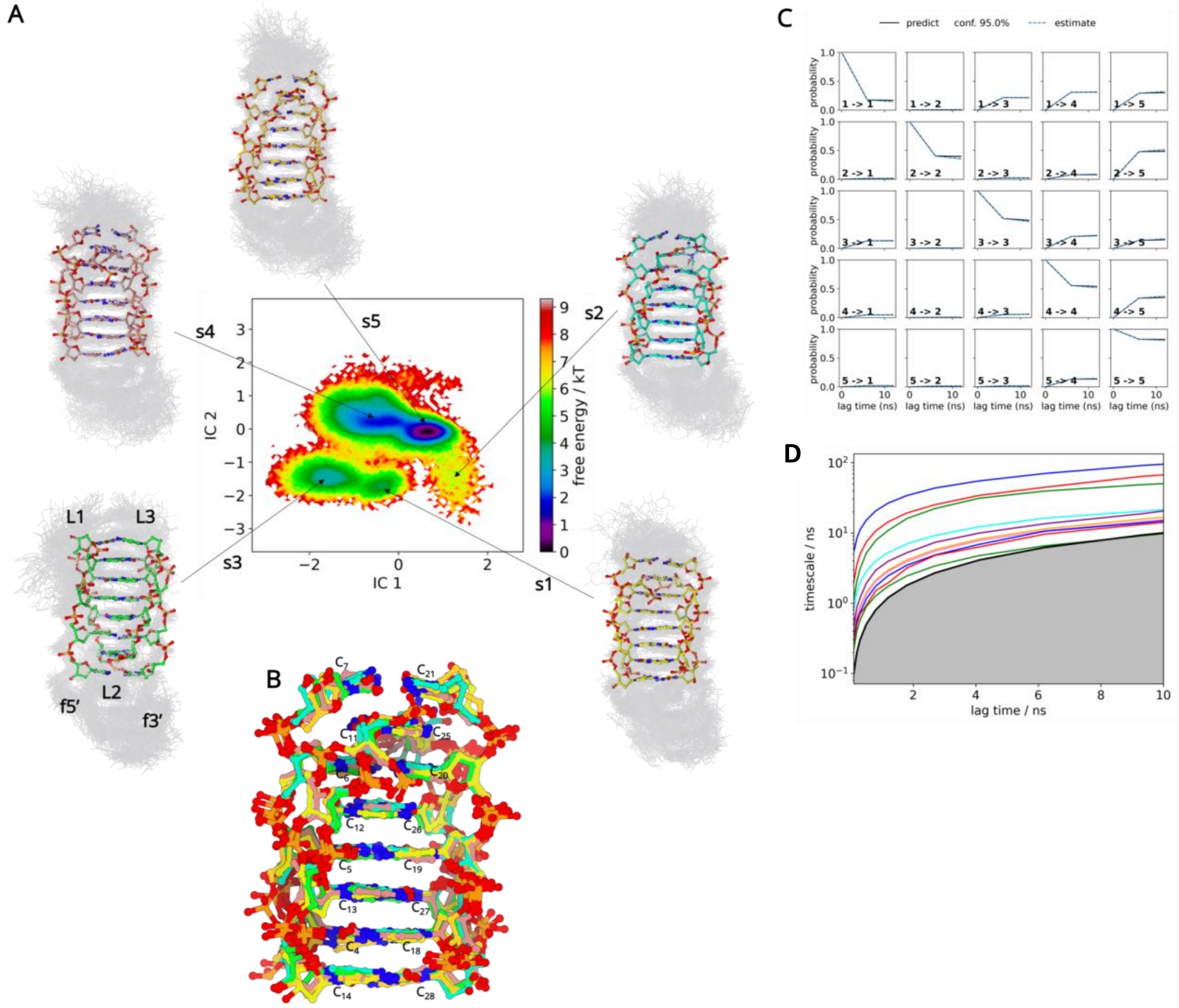

Supplementary Figure 20: (A) Projection of the Markov state model free energy landscape along the time-lagged independent components IC1 and IC2 of 4C. The representative structures s1, s2, s3, s4 and s5 derived from the dynamics of the central stem are marked in their basins. The positions of the loops have been marked L1, L2 and L3, while that of the 5' and 3' flanking nucleotides are labelled f5' and f3' respectively. (B) Superimposition of the five representative central stem structures of 4Cdel. (C) A Chapman-Kolmogorov (CK) plot is used to validate the self-consistency of the built MSM. 5 macrostates were defined. (D) The implied timescale (ITS) plot.

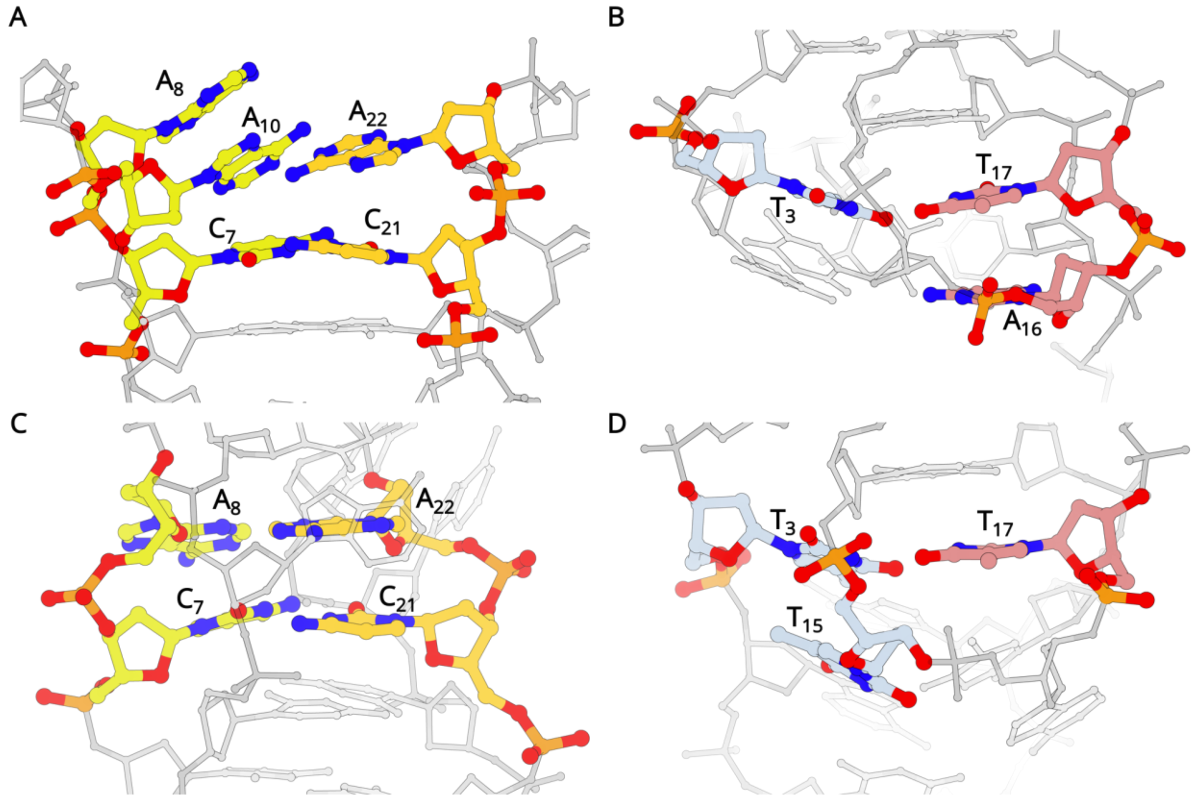

Supplementary Figure 21: Sampling the crystallographic conformations in 4Cdel (A) Loops 1 (yellow) and 3 (orange). The non-canonical A_{10}:A_{22} base pair caps the terminal C_7:C_{21} base pair. A_8 makes π-stack with A_{10}; (B) Loop 2 (pink) and the flanking (cyan) nucleotides in 4Cdel. A_{16} π-stacks below T_{17} stabilising the T_3:T_{17} base pair. The crystallographic conformations observed in 4C (C) The π-stacking of two non-canonical base pairs of A_8:A_{22} above C_7:C_{21} in Loops 1 and 3 and (D) Loop 2 and the flanking bases, where T_{15} stacks below T_3 in 4C.

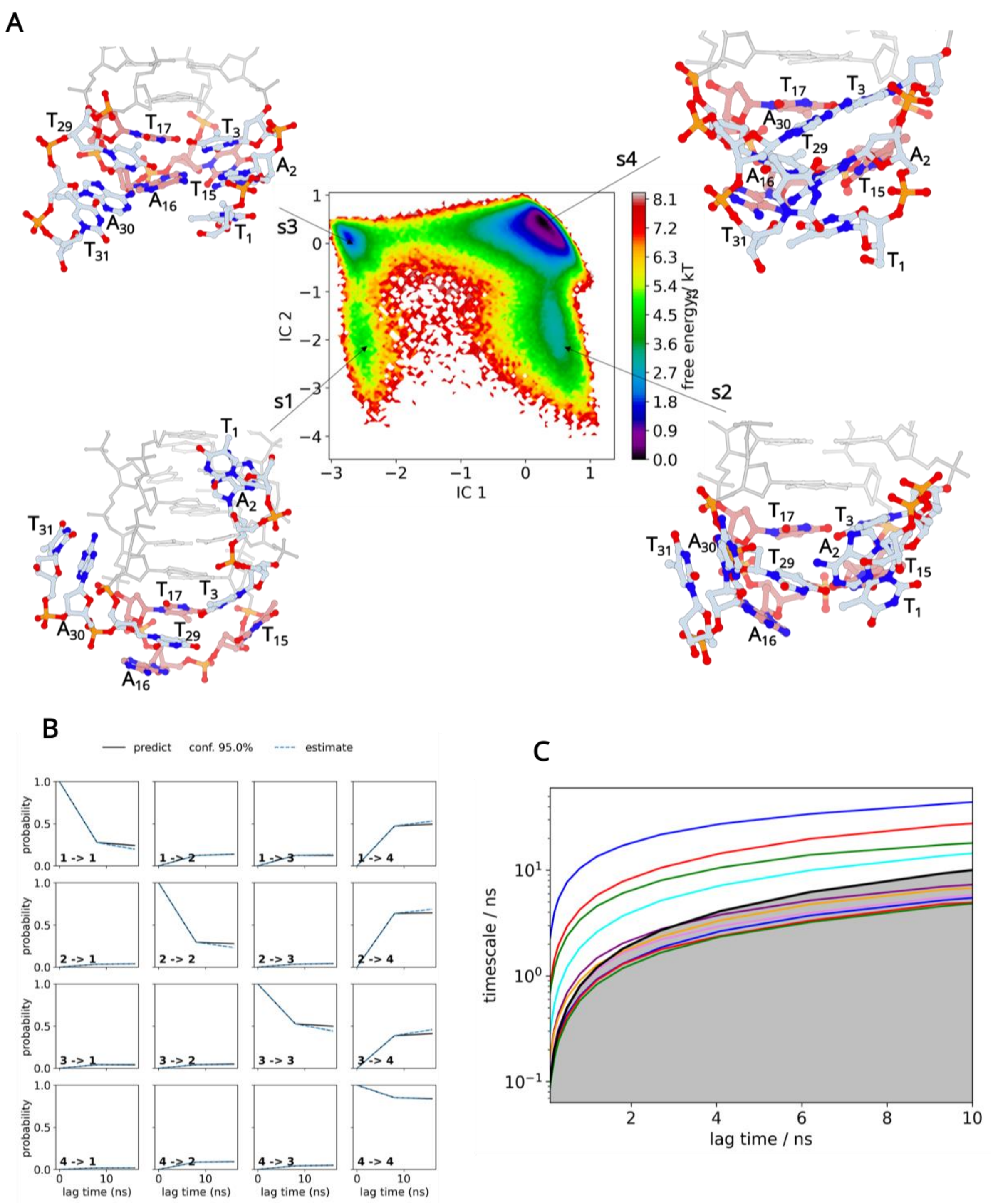

Supplementary Figure 22: (A) The free energy landscape of Loop 2 (pink) and the 5′ and 3′ flanking nucleotides (cyan) in the 4C. Four structures were identified that represented their respective clusters. In the most stable conformation (s4), loop 2 interacts with the flanking region to generate a stable conformation. The extra nucleotide in the 3′ flanking region enhances interactions to further stabilise the i-motif. (B) The Chapman-Kolmogorov test and (C) the implied time scale plots.

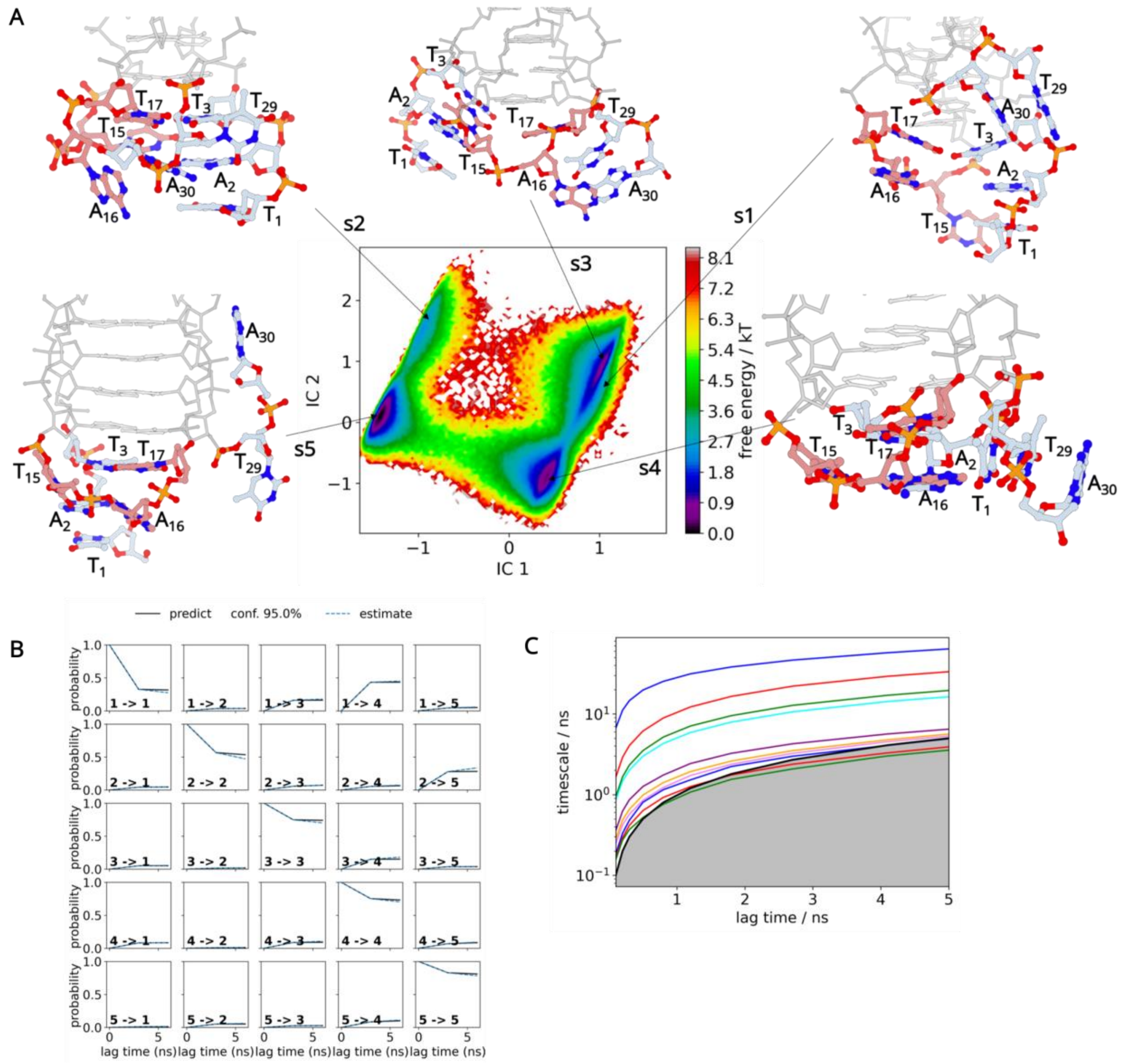

Supplementary Figure 23: (A) The free energy landscape of Loop 2 (pink) and the 5’ and 3’ flanking nucleotides (cyan) in the 4Cdel. Five structures were identified that represented their respective clusters. In the most stable conformation (s4), loop 2 interacts with the flanking region to generate a stable conformation. (B) The Chapman-Kolmogorov test and (C) the implied time scale plots.

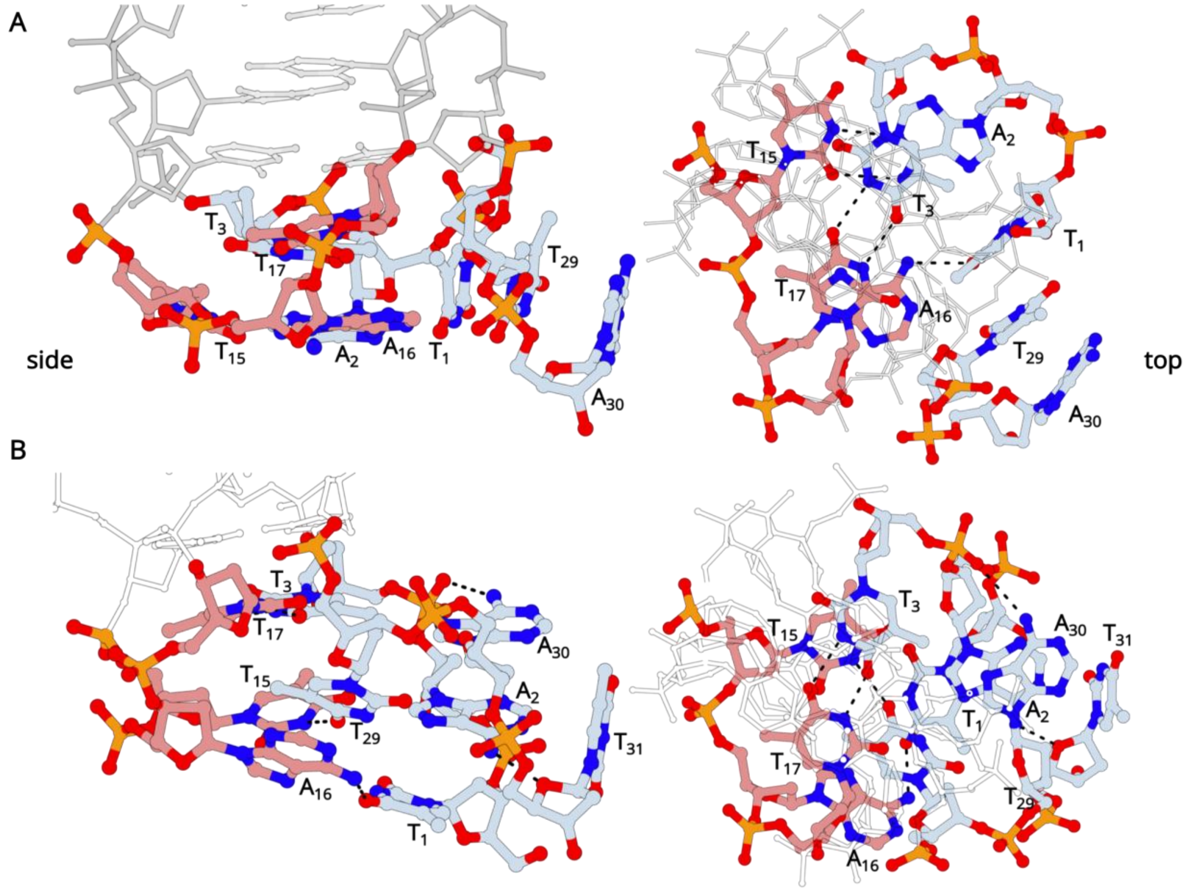

Supplementary Figure 24: Loop2 interactions with the 5'/3' flanking nucleotides in (A) 4Cdel and (B) 4C. In 4Cdel, one nucleotide is less in the 3' flank, in addition to the T_{13}:T_{17} base pair, A_{16} makes hydrogen bond with T_1, T_{15} with A_2. T_1 from the 5' flank is tilted inwards forming a 3-base π-stack with T_{29} and T_{30} from the 3' flanking nucleotides. In 4C, an extra nucleotide in the 3' flank results in enhanced hydrogen bonding and π-stacking interactions leading to a more structured capping of the end of the 4C i-motif. The side and top views if the interactions have been illustrated of Loop 2 (pink) and flanking nucleotides (cyan).

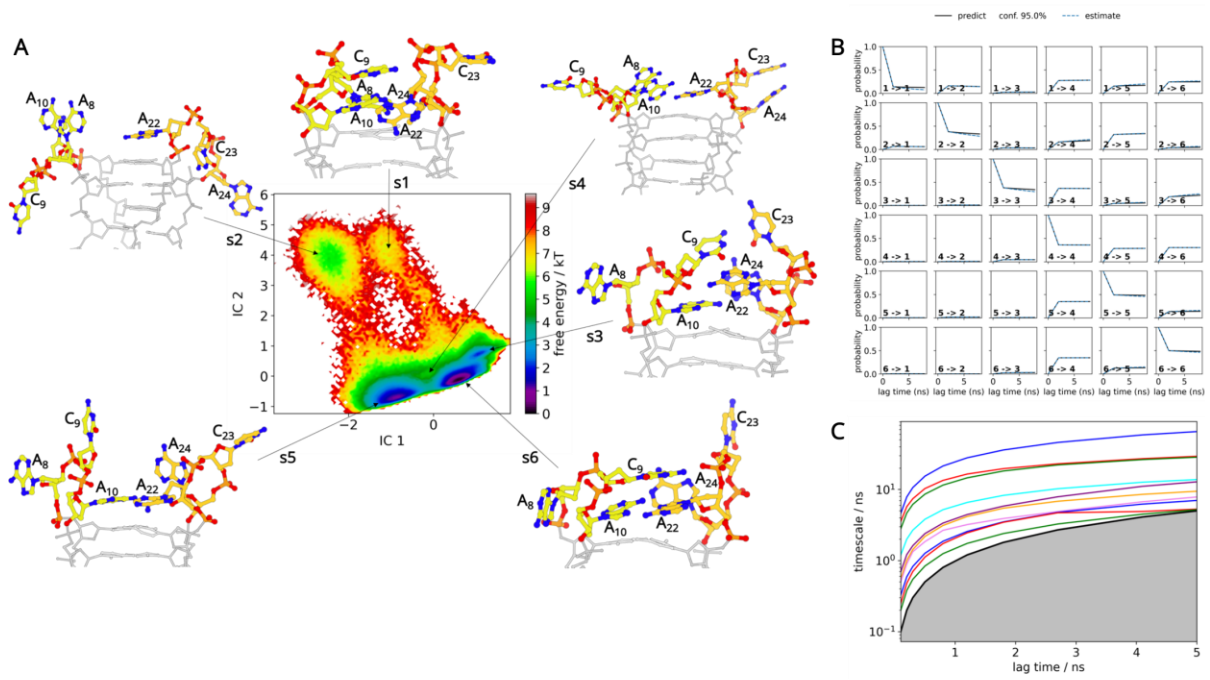

Supplementary Figure 25: (A) The free energy landscape of Loop 1 (yellow) and Loop 3 (orange) in 4Cdel structure. Six structures were identified that represented their respective clusters. Two of these (s5 and s6) correspond to low energy conformation. In the most stable conformation (s6), C9 from Loop 1 stacks on top of A_{22} to stabilise the A_{10}:A_{22} non-canonical base pair. (B) The Chapman-Kolmogorov test and (C) the implied time scale plots.

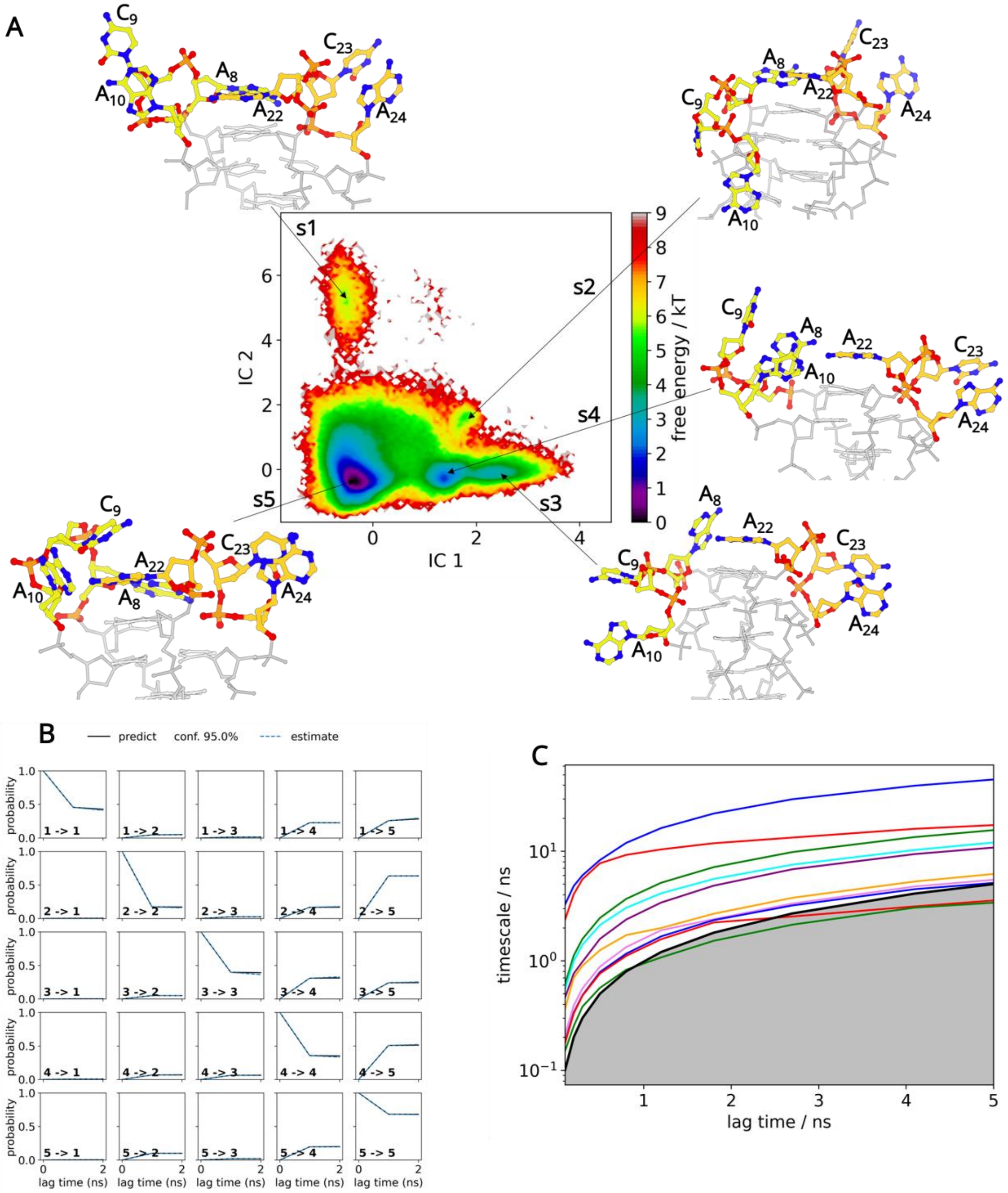

Supplementary Figure 26: (A) The free energy landscape of Loop 1 (yellow) and Loop3 (orange) in the 4C structure. Five structures were identified that represented their respective clusters. s5 represents the most stable state where A8:A22 base pair is formed while C_9 from loop 1 makes π-stacking interactions with A_8. Loop 3 is stabilised by π-stacking interactions between C_{23} and A_{24}. (B) The Chapman-Kolmogorov test and (C) the implied time scale plots.

3) SUPPLEMENTARY TABLES

Supplementary Table 1. The sequences of the 4C analogues with different flanking sequences and brominated sequence used for crystallisation screenings.

4C analogues	Sequence (5' → 3')
**4C**	TATCCCCACACCCCTATCCCCACACCCCTAT
**4Ca**	TCCCCACACCCCTATCCCCACACCCCT
**4Cb**	ATCCCCACACCCCTATCCCCACACCCC
**4C-Br**	TAT^{Br}CCCCACACCCCT^{Br}ATCCCCACACCCCTAT

Supplementary Table 2. Crystallisation conditions for obtaining crystals of each sequence.

Sequence name	[DNA solution] (mM)	Crystallisation solution components	Drop: (µL DNA solution + µL crystallisation solution)	Crystallisation solution equilibrated against
4C	0.3	49 mM sodium cacodylate pH 5.5<br>45 mM NaCl<br>23 % v/v MPD<br>2.9 mM spermine	1.2 + 1	49 mM sodium cacodylate pH 5.5<br>45 mM NaCl<br>23 % v/v MPD<br>2.9 mM spermine
4C - Br	0.3	49 mM sodium cacodylate pH 5.5<br>45 mM NaCl<br>23 % v/v MPD<br>2.9 mM spermine	1+1	49 mM sodium cacodylate pH 5.5<br>45 mM NaCl<br>20 % v/v MPD<br>2.9 mM spermine
4Ca	0.75	49 mM sodium cacodylate pH 5.5<br>45 mM NaCl<br>2.2 M ammonium sulfate<br>2.9 mM spermine<br>10% v/v glycerol<br><br>Or<br><br>49 mM sodium cacodylate pH 5.5<br>45 mM NaCl<br>23 % v/v MPD<br>2.9 mM spermine	1+1	49 mM sodium cacodylate pH 5.5<br>45 mM NaCl<br>2.2 M ammonium sulfate<br>2.9 mM spermine<br>10% v/v glycerol<br><br>Or<br><br>49 mM sodium cacodylate pH 5.5<br>45 mM NaCl<br>15 % v/v MPD<br>2.9 mM spermine
4Cb	0.75	49 mM sodium cacodylate pH 5.5<br>45 mM NaCl<br>23 % v/v MPD<br>2.9 mM spermine	1+1	49 mM sodium cacodylate pH 5.5<br>45 mM NaCl<br>17-21 % v/v MPD<br>2.9 mM spermine

Supplementary Table 3. Data collection and refinement statistics for 4C.

Values in parentheses are for the highest-resolution shell.

**Sequence name**	**4C**
**Sequence**	TATCCCCACACCCCTATCCCCACACCCCTAT
**Data collection**	
**Beamline**	Diamond Light Source I23
**Wavelength (Å)**	2.4797
**Space group**	$P2_12_12_1$
**Unit cell dimensions**<br>***a, b, c (Å)***<br>***α, β, γ (°)***	 47.41 51.51 69.85<br>90.00 90.00 90.00
I / σ I	30.40 (1.7)
**R_{meas} (within I+/I-)**<br>**R_{meas} (all I+ & I-)**	0.165 (2.009)<br>0.169 (2.014)
**R_{merge} (within I+/I-)**<br>**R_{merge} (all I+ & I-)**	0.162 (1.920)<br>0.167 (1.966)
**Completeness (%)**	95.2 (88.0)
**Multiplicity**	44.5 (22.7)
**Resolution range (Å)**	47.42 – 2.25 (2.32 – 2.25)
**Total number of reflections**	362297 (14886)
**No. of unique reflections**	8133 (657)
**$CC_{1/2}$**	0.991 (0.748)
**Anomalous completeness %**	94.6 (86.5)
**Anomalous multiplicity**	23.3 (11.9)
**DelAnom correlation between half-sets** (inner shell)	0.537
**Refinement**	
**Completeness (working + test) (%)**	93.14 (89.08)
**Resolution range (Å)**	41.46-2.25 (2.58-2.25)
**No. of unique reflections refined against**	7957 (2331)
**No. of molecules in the ASU**	2
**R_{free}/R_{work}**	0.2948/0.2487 (0.4622/0.3748)
**No. of atoms**<br>**DNA (in the asymmetric unit)**<br>**DNA (per chain)**<br>**Water (in the asymmetric unit)**	 1212<br>606<br>22
**Mean B value (overall Å^2)**	80.06
**RMSD from ideal values**<br>**Bond lengths (Å)**<br>**Bond angles (°)**	 0.013<br>1.113

Supplementary Table 4. Data collection and processing statistics for 4C-Br.

Values in parentheses are for the highest-resolution shell.

Sequence name	4C – Br		
Sequence	5' – TAT^{Br}**C**CCCACACCCCT^{Br}**A**TCCCCACACCCCTAT – 3'		
Beamline	Diamond Light Source I03		
Wavelength (Å)	0.9196	0.9203	0.9117
Space group	$P2_12_12_1$	$P2_12_12_1$	$P2_12_12_1$
Unit cell dimensions<br>*a, b, c (Å)*<br>*α, β, γ (°)*	 47.18 51.57 69.94<br>90.00 90.00 90.00	 47.19 51.57 69.89<br>90.00 90.00 90.00	 47.19 51.58 69.87<br>90.00 90.00 90.00
I / σ I	18.7 (2.6)	21.9 (3.6)	22.3 (3.9)
R_{meas} (within I+/I-)<br>R_{meas} (all I+ & I-)	0.065 (1.085)<br>0.067 (1.083)	0.058 (0.764)<br>0.058 (0.763)	0.058 (0.697)<br>0.062 (0.697)
R_{merge} (within I+/I-)<br>R_{merge} (all I+ & I-)	0.060 (1.008)<br>0.064 (1.043)	0.054 (0.709)<br>0.055 (0.734)	0.054 (0.646)<br>0.060 (0.670)
Completeness (%)	98.9 (98.0)	98.9 (98.2)	98.9 (98.3)
Multiplicity	13.2 (13.7)	13.2 (13.7)	13.2 (13.7)
Resolution range (Å)	51.57 – 2.38<br>(2.47 – 2.38)	51.57 – 2.38<br>(2.47 – 2.38)	51.58 – 2.38<br>(2.47 – 2.38)
Total number of reflections	94750 (10092)	94731 (10133)	94770 (10158)
No. of unique reflections	7152 (734)	7152 (738)	7153 (740)
$CC_{1/2}$	1.000 (0.897)	1.000 (0.938)	1.000 (0.857)
Anomalous completeness %	99.3 (98.5)	99.3 (98.6)	99.3 (98.6)
Anomalous multiplicity	7.2 (7.3)	7.2 (7.3)	7.3 (7.3)
DelAnom correlation between half-sets (inner shell)	0.808	0.351	0.920

Supplementary Table 5. Narrow and wide groove dimensions based on P – P distances of adjacent strands. Average dimensions for each groove are shown in the schematic in Supplementary Figure 12 with P – P distances of nucleotides from the cytosine rich core, loop or flank regions included in the calculation.

Groove	Nucleotides	P – P distance (Å)		Region
		Strand A	Strand B	
Narrow groove 1	$C_4 – T_{15}$	9.23	8.25	Core – Loop 2
	$C_5 – C_{14}$	6.32	6.58	Core
	$C_6 – C_{13}$	7.20	7.06	Core
	$C_7 – C_{12}$	6.97	6.73	Core
	$A_8 – C_{11}$	6.87	7.65	Loop 1 – Core
	$C_9 – A_{10}$	5.88	6.17	Loop 1
Narrow groove 2	$C_{18} – T_{29}$	7.64	6.52	Core – Flank 2
	$C_{19} – C_{28}$	6.60	8.39	Core
	$C_{20} – C_{27}$	6.96	6.76	Core
	$C_{21} – C_{26}$	5.80	5.98	Core
	$A_{22} – C_{25}$	9.36	7.98	Loop 3 – Core
	$C_{23} – A_{24}$	5.45	6.96	Loop 3
Wide groove 1	$A_{10} – A_{22}$	14.37	21.10	Loop 1 – Loop 3
	$C_{11} – C_{21}$	12.20	13.41	Core
	$C_{12} – C_{20}$	12.35	14.03	Core
	$C_{13} – C_{19}$	12.53	13.50	Core
	$C_{14} – C_{18}$	12.50	13.75	Core
	$T_{15} – T_{17}$	11.31	12.13	Loop 2 – Loop 2
Wide groove 2	$C_4 – T_{29}$	17.96	18.05	Core – Flank 2
	$C_5 – C_{28}$	17.06	16.56	Core
	$C_6 – C_{27}$	16.47	16.66	Core
	$C_7 – C_{26}$	16.55	16.54	Core
	$A_8 – C_{25}$	17.27	15.90	Loop 1 – Core
	$C_9 – A_{24}$	20.58	20.47	Loop 1 – Loop 3

Supplementary Table 6. Intramolecular interactions (within each strand) of the 4C i-motif based on the crystal structure.

5' – $T_1A_2T_3C_4C_5C_6C_7A_8C_9A_{10}C_{11}C_{12}C_{13}C_{14}T_{15}A_{16}T_{17}C_{18}C_{19}C_{20}C_{21}A_{22}C_{23}A_{24}C_{25}C_{26}C_{27}C_{28}T_{29}A_{30}T_{31}$ – 3'

Flank 1 ($T_1A_2T_3$) | Loop 1 ($A_8C_9A_{10}$) | Loop 2 ($T_{15}A_{16}T_{17}$) | Loop 3 ($A_{22}C_{23}A_{24}$) | Flank 2 ($T_{29}A_{30}T_{31}$)

**Mismatched TT and AA base pairs**			
Strand	Nucleotides and atoms involved	Distance (Å)	Type of interaction
**A**	T_{17} (N_3) Loop 2 – T_3 (O_4) Flank 1<br>T_{17} (O_4) Loop 2 – T_3 (N_3) Flank 1	3.2<br>3.0	Hydrogen bonds
	A_{10} (N_6) Loop 1 – A_{22} (N_1) Loop 3<br>A_{10} (N_7) Loop 1 – A_{22} (N_6) Loop 3	2.7<br>2.9	Hydrogen bonds
**B**	T_{17} (N_3) Loop 2 – T_3 (O_4) Flank 1<br>T_{17} (O_4) Loop 2 – T_3 (N_3) Flank 1	2.9<br>2.8	Hydrogen bonds
	A_8 (N_6) Loop 1 – A_{22} (N_1) Loop 3<br>A_{22} (N_6) Loop 3 – A_8 (N_7) Loop 1	2.7<br>3.6	Hydrogen bonds
**AT interactions**			
Strand	Nucleotides and atoms involved	Distance (Å)	Type of interaction
**A**	A_{16}(N_6) Loop 2 – T_{15}(O_2) Loop 2	3.1	Hydrogen bond
**π–π stacking**			
Strand	Nucleotides involved	Distance (Å)	Type of interaction
**A**	A_8 Loop 1 – A_{10} Loop 1	-	π–π stacking
	A_{10} Loop 1 – C_7	-	π–π stacking
	A_{16} Loop 2 – T_{17} Loop 2	-	π–π stacking
	T_{29} Flank 2 – A_{30} Flank 2	-	π–π stacking
**B**	C_7 – A_8 Loop 1	-	π–π stacking
	C_{21} – A_{22} Loop 3	-	π–π stacking
**Interactions between nucleotides and the phosphate backbone**			
Strand	Nucleotides involved	Distance (Å)	Type of interaction
**B**	A_{16}(N_6) Loop 2 – A_{24} (OP1) Loop 3<br>A_{16}(N_6) Loop 2 – A_{24} (OP2) Loop 3	2.8<br>3.1	Hydrogen bonds<br>(A_{16} is disordered)

Supplementary Table 7. Intermolecular interactions between strands A and B of the 4C i-motif based on the crystal structure.

5' – $T_1A_2T_3C_4C_5C_6C_7A_8C_9A_{10}C_{11}C_{12}C_{13}C_{14}T_{15}A_{16}T_{17}C_{18}C_{19}C_{20}C_{21}A_{22}C_{23}A_{24}C_{25}C_{26}C_{27}C_{28}T_{29}A_{30}T_{31}$ – 3'

Flank 1 (T1A2T3) **Loop 1** (A8C9A10) **Loop 2** (T15A16T17) **Loop 3** (A22C23A24) **Flank 2** (T29A30T31)

**Mismatched AA base pairs**			
**Strand A** nucleotides and atoms involved	**Strand B** nucleotides and atoms involved	Distance (Å)	Type of interaction
A_2 (N_6) Flank 1<br>A_2 (N_1) Flank 1	A_{24} (N_7) Loop 3<br>A_{24} (N_6) Loop 3	2.8<br>2.7	Hydrogen bonds
A_{24} (N_6) Loop 3<br>A_{24} (N_7) Loop 3	A_2 (N_1) Flank 1<br>A_2 (N_6) Flank 1	3.0<br>3.1	Hydrogen bonds
**AT base pairs**			
**Strand A** nucleotides and atoms involved	**Strand B** nucleotides and atoms involved	Distance (Å)	Type of interaction
A_8 (N_6) Loop 1<br>A_8 (N_1) Loop 1<br><br>A_8 (N_6) Loop 1<br>A_8 (N_7) Loop 1	T_{15} (O_2) Loop 2<br>T_{15} (N_3) Loop 2<br><br>T_{29} (O_4) Flank 2<br>T_{29} (N_3) Flank 2	3.2<br>2.8<br><br>3.0<br>2.9	**TAT triad**<br>Hydrogen bonds<br><br>Hoogsteen base pairing
T_{15} (O_4) Loop 2<br>T_{15} (N_3) Loop 2	A_{10} (N_6) Loop 1<br>A_{10} (N_1) Loop 1	3.0<br>3.0	Watson and Crick base pairing
**CC base pairs**			
**Strand A** nucleotides and atoms involved	**Strand B** nucleotides and atoms involved	Distance (Å)	Type of interaction
C_9 (N_4) Loop 1<br>C_9 (N_3) Loop 1<br>C_9 (O_2) Loop 1	C_{23}(O_2) Loop 3<br>C_{23}(N_3) Loop 3<br>C_{23} (N_4) Loop 3	2.9<br>2.8<br>2.9	Hydrogen bonds
C_{23}(O_2) Loop 3<br>C_{23}(N_3) Loop 3<br>C_{23} (N_4) Loop 3	C_9 (N_4) Loop 1<br>C_9 (N_3) Loop 1<br>C_9 (O_2) Loop 1	2.9<br>2.8<br>2.9	Hydrogen bonds
**π–π stacking**			
**Strand A** nucleotides and atoms involved	**Strand B** nucleotides and atoms involved	Distance (Å)	Type of interaction
A_{24} Loop 3	T_1 Flank 1	-	π–π stacking
T_1 Flank 1	A_{24} Loop 3	-	π–π stacking
A_2 Flank 1	C_9 Loop 1	-	π–π stacking
T_{15} Loop 2	T_{31} Flank 2	-	π–π stacking<br>(T_{31} is disordered)
A_{16} Loop 2	A_{22} Loop 3	-	π–π stacking
A_{22} Loop 3	T_{15} Loop 2	-	π–π stacking
C_9 Loop 1	A_2 Flank 1	-	π–π stacking

Supplementary Table 8. Nucleotide backbone torsion angles and sugar puckers in the two strands of the 4C i – motif calculated by the 3DNA.[8] Δ_{A-B} = A-B , Δ_{A-B}*= absolute angle difference between strand A and B within the range of 0° to 180°.

Base	Strand	Alpha	Beta	Gamma	Delta	Epsilon	Zeta	Chi	Orientation	Puckering
T1	A	---	---	70.8	149.2	-98.6	78.9	-133	anti	C2' - endo
	B	---	---	-40.3	159	-171.7	145.7	-142.6	anti	C3' - exo
	Δ_{A-B}	---	---	111.1	-9.8	73.1	-66.8	9.6		
	Δ_{A-B}*	---	---	111.1	9.8	73.1	66.8	9.6		
A2	A	-161.3	-143.1	68.4	102.3	-85.1	178.5	-77.4	syn	C2' - exo
	B	-58.5	136.6	47.8	112.5	-104.1	-152.3	-71.3	syn	C2' - exo
	Δ_{A-B}	-102.8	-279.7	20.6	-10.2	19	330.8	-6.1		
	Δ_{A-B}*	102.8	80.3	20.6	10.2	19	29.2	6.1		
T3	A	-35.3	-166.4	41.6	145.8	-129	-80.1	-146.3	anti	C3' - exo
	B	-24.3	-163.9	44.2	148.8	-118.9	-65.3	-158.9	anti	C3' - exo
	Δ_{A-B}	-11	-2.5	-2.6	-3	-10.1	-14.8	12.6		
	Δ_{A-B}*	11	2.5	2.6	3	10.1	14.8	12.6		
C4	A	-58.5	169.6	52.7	128.3	-126	-121.6	-106.7	anti	C2' - endo
	B	-60.4	166.6	37.5	94.7	-166	-109.4	-120.7	anti	C4' - exo
	Δ_{A-B}	1.9	3	15.2	33.6	40	-12.2	14		
	Δ_{A-B}*	1.9	3	15.2	33.6	40	12.2	14		
C5	A	67.8	-158.1	-112.6	130.7	-161.5	-111.8	-92.1	anti	C2' - endo
	B	142.1	-162.8	-151.9	116.2	-131	-126.5	-124.4	anti	C1' - exo
	Δ_{A-B}	-74.3	4.7	39.3	14.5	-30.5	14.7	32.3		
	Δ_{A-B}*	74.3	4.7	39.3	14.5	30.5	14.7	32.3		
C6	A	174.4	174.1	163.5	84.9	67.6	75.3	-124.1	anti	C4' - exo
	B	94.6	-150.6	-130.6	106.8	-149.6	-41	-127.5	anti	C1' - exo
	Δ_{A-B}	79.8	324.7	294.1	-21.9	217.2	116.3	3.4		
	Δ_{A-B}*	79.8	35.3	65.9	21.9	142.8	116.3	3.4		
C7	A	97.1	163.9	174.5	141.1	-103.2	-119.8	-103.7	anti	C2' - endo
	B	-142.1	84	-171.4	136.3	-127.8	-139.5	-122	anti	C2' - endo
	Δ_{A-B}	239.2	79.9	345.9	4.8	24.6	19.7	18.3		
	Δ_{A-B}*	120.8	79.9	14.1	4.8	24.6	19.7	18.3		
A8	A	7	-114.1	-90.2	164.9	-149.3	-178.7	-98.8	anti	C3' - exo
	B	97.2	-155	-160.9	142	-96.8	150.6	64.8	syn	C2' - endo
	Δ_{A-B}	-90.2	40.9	70.7	22.9	-52.5	-329.3	-163.6		
	Δ_{A-B}*	90.2	40.9	70.7	22.9	52.5	30.7	163.6		
C9	A	62.1	174.7	53.6	94.8	-139.1	90	-139.2	anti	C3' - endo
	B	72.5	150.6	40.4	89.5	-174.9	109.6	-156.9	anti	C3' - endo
	Δ_{A-B}	-10.4	24.1	13.2	5.3	35.8	-19.6	17.7		
	Δ_{A-B}*	10.4	24.1	13.2	5.3	35.8	19.6	17.7		
A10	A	-91.6	-138.4	47.5	90.5	-173.7	-100.8	-114.5	anti	C4' - exo
	B	-62	143.5	42.6	110.5	-142.8	168.2	-134.3	anti	C1' - exo
	Δ_{A-B}	-29.6	-281.9	4.9	-20	-30.9	-269	19.8		
	Δ_{A-B}*	29.6	78.1	4.9	20	30.9	91	19.8		
C11	A	-49.5	173.1	55.6	90.1	-152.6	-92.5	-128.6	anti	C3' - endo
	B	-47.2	170.9	46	91.6	-122.5	-114	-140.5	anti	C3' - endo
	Δ_{A-B}	-2.3	2.2	9.6	-1.5	-30.1	21.5	11.9		
	Δ_{A-B}*	2.3	2.2	9.6	1.5	30.1	21.5	11.9		
C12	A	-49.2	171.5	54.2	85.7	-172.8	-73.7	-126.4	anti	C3' - endo
	B	-27.5	148.4	39.1	80.2	-160.8	-101.7	-124.9	anti	C3' - endo
	Δ_{A-B}	-21.7	23.1	15.1	5.5	-12	28	-1.5		
	Δ_{A-B}*	21.7	23.1	15.1	5.5	12	28	1.5		
C13	A	-86.4	-166.6	74.6	81.9	-153	-90.5	-122.9	anti	C3' - endo
	B	-42.7	158.1	59.1	81.7	-157.6	-90	-132.9	anti	C3' - endo
	Δ_{A-B}	-43.7	-324.7	15.5	0.2	4.6	-0.5	10		
	Δ_{A-B}*	43.7	35.3	15.5	0.2	4.6	0.5	10		
C14	A	-47.3	167	57.5	79.4	-145.8	-15.9	-120.2	anti	C3' - endo
	B	146.4	-172.8	-158.5	90.7	-113.6	-56	-125.2	anti	C3' - endo

	Δ_{A-B}	-193.7	339.8	216	-11.3	-32.2	40.1	5		
	Δ_{A-B}*	166.3	20.2	144	11.3	32.2	40.1	5		
T15	A	177.9	117.4	77.6	121.1	-106.8	-143.5	-175.4	anti	C1' - exo
	B	153	-166.1	77.1	145.5	-147.4	-160.1	-142.1	anti	C2' - endo
	Δ_{A-B}	24.9	283.5	0.5	-24.4	40.6	16.6	-33.3		
	Δ_{A-B}*	24.9	76.5	0.5	24.4	40.6	16.6	33.3		
A16	A	-173.2	-160.6	77.7	89.8	-137.6	-66.6	-174.5	anti	C3' - endo
	B	58.9	121.4	168.4	147.7	19.4	-173.5	69.2	syn	C3' - exo
	Δ_{A-B}	-232.1	-282	-90.7	-57.9	-157	106.9	-243.7		
	Δ_{A-B}*	127.9	78	90.7	57.9	157	106.9	116.3		
T17	A	-65.5	-177.2	49.5	86.8	-133.9	-87.5	-154.2	anti	C3' - endo
	B	-29.6	136.9	11.5	85.1	-140.2	-89.6	-144.1	anti	C3' - endo
	Δ_{A-B}	-35.9	-314.1	38	1.7	6.3	2.1	-10.1		
	Δ_{A-B}*	35.9	45.9	38	1.7	6.3	2.1	10.1		
C18	A	-55	-179.7	55.5	89.1	-167.6	-78.7	-125.2	anti	C3' - endo
	B	-45.2	178	52.6	91.8	-163.2	-91.6	-126	anti	C3' - endo
	Δ_{A-B}	-9.8	-357.7	2.9	-2.7	-4.4	12.9	0.8		
	Δ_{A-B}*	9.8	2.3	2.9	2.7	4.4	12.9	0.8		
C19	A	-68.8	-167.5	61.7	89.9	-162.5	-89.6	-118.7	anti	C3' - endo
	B	-53.9	179.9	51	87.3	-170.3	-80.4	-115.1	anti	C3' - endo
	Δ_{A-B}	-14.9	-347.4	10.7	2.6	7.8	-9.2	-3.6		
	Δ_{A-B}*	14.9	12.6	10.7	2.6	7.8	9.2	3.6		
C20	A	-63.7	170.7	69	86.5	-158.3	-84.9	-129	anti	C3' - endo
	B	-64.5	179.8	66.1	85.5	-149	-111.2	-120.4	anti	C3' - endo
	Δ_{A-B}	0.8	-9.1	2.9	1	-9.3	26.3	-8.6		
	Δ_{A-B}*	0.8	9.1	2.9	1	9.3	26.3	8.6		
C21	A	-44.4	-171.3	49.7	95.7	-144.1	-124.1	-103.3	anti	C4' - exo
	B	79.3	-141.6	-85.7	162.1	-115.9	-127.2	-97.9	anti	C3' - exo
	Δ_{A-B}	-123.7	-29.7	135.4	-66.4	-28.2	3.1	-5.4		
	Δ_{A-B}*	123.7	29.7	135.4	66.4	28.2	3.1	5.4		
A22	A	-42.9	166.6	48.4	155.7	-98.1	96.3	-55.1	syn	C3' - exo
	B	81.4	-138.9	-158.5	144.8	-93.5	134.7	-92.8	anti	C2' - endo
	Δ_{A-B}	-124.3	305.5	206.9	10.9	-4.6	-38.4	37.7		
	Δ_{A-B}*	124.3	54.5	153.1	10.9	4.6	38.4	37.7		
C23	A	73	177.7	28.4	89.3	-153.7	-90.5	-147.2	anti	C3' - endo
	B	39.3	165.1	74.9	140.2	-122.7	-40.9	-142	anti	C2' - endo
	Δ_{A-B}	33.7	12.6	-46.5	-50.9	-31	-49.6	-5.2		
	Δ_{A-B}*	33.7	12.6	46.5	50.9	31	49.6	5.2		
A24	A	-49.1	-144.5	40.8	145	-103.7	-67.5	-90.8	anti	C2' - endo
	B	-70.6	-123.2	-70.1	165.3	-109.5	-92.6	-66.9	syn	C3' - exo
	Δ_{A-B}	21.5	-21.3	110.9	-20.3	5.8	25.1	-23.9		
	Δ_{A-B}*	21.5	21.3	110.9	20.3	5.8	25.1	23.9		
C25	A	-62.3	174.4	173.3	138.4	-153.1	-56.3	-137.1	anti	C2' - endo
	B	62.5	159.2	53.7	141.2	-153.6	-66.7	-108.5	anti	C2' - endo
	Δ_{A-B}	-124.8	15.2	119.6	-2.8	0.5	10.4	-28.6		
	Δ_{A-B}*	124.8	15.2	119.6	2.8	0.5	10.4	28.6		
C26	A	154.4	142	-177.1	118.3	-156.9	-72.7	-109.6	anti	C1' - exo
	B	167.7	135.1	176.9	120.5	-161.6	-73.8	-106.9	anti	C1' - exo
	Δ_{A-B}	-13.3	6.9	-354	-2.2	4.7	1.1	-2.7		
	Δ_{A-B}*	13.3	6.9	6	2.2	4.7	1.1	2.7		
C27	A	-157.5	122.3	171	128.2	-150.2	-75.8	-113.5	anti	C2' - endo
	B	179.1	138.3	170.1	91	-135.7	-72.6	-116.7	anti	C4' - exo
	Δ_{A-B}	-336.6	-16	0.9	37.2	-14.5	-3.2	3.2		
	Δ_{A-B}*	23.4	16	0.9	37.2	14.5	3.2	3.2		
C28	A	-175.4	134.9	169.9	112.7	-88.6	134.9	-108.2	anti	C1' - exo
	B	-104.7	98.2	165.3	136.3	-113.2	-68.3	-139	anti	C2' - endo
	Δ_{A-B}	-70.7	36.7	4.6	-23.6	24.6	203.2	30.8		
	Δ_{A-B}*	70.7	36.7	4.6	23.6	24.6	156.8	30.8		
T29	A	3.1	123.3	9.1	150.5	-167.2	-136.3	-107.8	anti	C2' - endo
	B	-56.5	-128.1	-62.1	136.2	-151.5	-118.4	88	syn	C2' - endo
	Δ_{A-B}	59.6	251.4	71.2	14.3	-15.7	-17.9	-195.8		
	Δ_{A-B}*	59.6	108.6	71.2	14.3	15.7	17.9	164.2		

A30	A	16	129.3	16.3	140.9	-179	163	-86	syn	C2' - endo
	B	41.7	-122.9	-100.4	70.9	-149.8	171.6	-159.7	anti	C3' - endo
	Δ_{A-B}	-25.7	252.2	116.7	70	-29.2	-8.6	73.7		
	Δ_{A-B}*	25.7	107.8	116.7	70	29.2	8.6	73.7		
T31	A	15.5	113.5	8.3	89.9	---	---	-90.9	anti	C3' - endo
	B	36.8	122.7	-8.7	150.1	---	---	-151.9	anti	C2' - endo
	Δ_{A-B}	-21.3	-9.2	17	-60.2	---	---	61		
	Δ_{A-B}*	21.3	9.2	17	60.2	---	---	61		

Supplementary Table 9. Summary of the simulation set up.

	**4C**	**4Cdel**
Simulation Box size (Å^3)	64.035 63.133 71.425	58.321 59.233 68.989
No. of water atoms	21378	17640
Total no. of atoms	22373	18600
Ionic Conc.	0.15 M	0.15 M
Total no. of trajectories	1058 x 50 ns	1208 x 50 ns

Supplementary Table 10. Description of key parameters for creating Markov State Models for sequences 4C and 4Cdel.

	**Features**	**tICA lag**	**N^o dimensions**	**Clusters**	**MSM lag**	**N^o states**
**4C stem**	α/γ dihedral + N3 distance of stem bases	450	3	200	60	5
**4C Loop 1 and 3**	α/γ dihedral + χ	30	3	200	10	5
**4C Loop 2**	α/γ dihedral + χ	30	3	200	20	4
**4Cdel stem**	α/γ dihedral + N3 distance of stem bases	400	5	200	60	3
**4Cdel Loop 1 and 3**	α/γ dihedral + χ	40	3	200	20	6
**4Cdel Loop 2**	α/γ dihedral + χ	40	3	200	30	5

4) SUPPLEMENTARY REFERENCES

1. D.A. Case RMB, D.S. Cerutti, T.E. Cheatham, III, T.A. Darden, R.E. Duke, T.J. Giese, H. Gohlke,, A.W. Goetz NH, S. Izadi, P. Janowski, J. Kaus, A. Kovalenko, T.S. Lee, S. LeGrand, P. Li, C., Lin TL, R. Luo, B. Madej, D. Mermelstein, K.M. Merz, G. Monard, H. Nguyen, H.T. Nguyen, I., Omelyan AO, D.R. Roe, A. Roitberg, C. Sagui, C.L. Simmerling, W.M. Botello-Smith, J. Swails,, R.C. Walker JW, R.M. Wolf, X. Wu, L. Xiao and P.A. Kollman. AMBER 2016. *University of California, San Francisco*, (2016).

2. Ivani I*, et al.* Parmbsc1: a refined force field for DNA simulations. *Nature Methods* **13**, 55-58 (2016).

3. Zgarbová M, Šponer J, Otyepka M, Cheatham TE, 3rd, Galindo-Murillo R, Jurečka P. Refinement of the Sugar-Phosphate Backbone Torsion Beta for AMBER Force Fields Improves the Description of Z- and B-DNA. *Journal of Chemical Theory and Computation* **11**, 5723-5736 (2015).

4. Pérez A, Herrera-Nieto P, Doerr S, De Fabritiis G. AdaptiveBandit: A Multi-armed Bandit Framework for Adaptive Sampling in Molecular Simulations. *Journal of Chemical Theory and Computation* **16**, 4685-4693 (2020).

5. Scherer MK*, et al.* PyEMMA 2: A Software Package for Estimation, Validation, and Analysis of Markov Models. *Journal of Chemical Theory and Computation* **11**, 5525-5542 (2015).

6. Kuroda A*, et al.* Insulin gene expression is regulated by DNA methylation. *PLoS One* **4**, e6953 (2009).

7. Zuker M. Mfold web server for nucleic acid folding and hybridization prediction. *Nucleic Acids Res.* **31**, 3406-3415 (2003).

8. Lu XJ, Olson WK. 3DNA: a software package for the analysis, rebuilding and visualization of three-dimensional nucleic acid structures. *Nucleic Acids Res.* **31**, 5108-5121 (2003).
